# When do book–tax differences matter for earnings persistence? The role of sales decline

**DOI:** 10.1371/journal.pone.0344270

**Published:** 2026-10-28

**Authors:** Dang Anh Tuan, Nguyen Ngoc Khanh Dung

**Affiliations:** School of Finance and Accounting, Industrial University of Ho Chi Minh City, Ho Chi Minh City, Vietnam; IU Internationale Hochschule GmbH, GERMANY

## Abstract

This study examines the association between book–tax differences (BTDs), sales decline, and earnings persistence among non-financial listed firms in Vietnam. Adopting a conditional perspective, the study treats BTDs as observable signals of accounting–tax divergence, reporting environments, and transitory earnings components, while sales decline is conceptualized as an adverse operating state that may weaken the association between current and future earnings. Using a balanced panel of 325 firms from 2013 to 2023, comprising 3,575 firm-year observations, earnings persistence is measured through the association between current and one-year-ahead pre-tax book income. Earnings are further decomposed into operating cash flows and accruals to examine component-level persistence. The empirical analysis uses OLS regressions with industry and year fixed effects and firm-clustered standard errors. Additional analyses control for earnings management and tax avoidance and test the robustness of the findings using a continuous measure of sales decline, a sample excluding the COVID-19 period, and an alternative annual-decile classification of large BTDs. The results show that pre-tax book income is persistent, but its persistence is significantly lower during periods of sales decline. The evidence for BTDs is asymmetric: large positive BTDs are more consistently associated with lower earnings persistence, whereas the evidence for large negative BTDs varies across model specifications and earnings components. The three-way interactions among sales decline, BTD classifications, and current earnings are not statistically significant in the main analyses, indicating that the evidence does not support a broad BTD-specific amplification mechanism. Overall, the findings suggest that earnings persistence is shaped by earnings components, accounting–tax reporting signals, and operating conditions. The study highlights the importance of interpreting BTDs jointly with sales trends and earnings components in the Vietnamese institutional setting.

## 1. Introduction

Earnings persistence is a central attribute of accounting information quality, indicating how much current earnings are informative about a firm’s future earnings performance. Within the literature on earnings attributes, book–tax differences, the gap between financial accounting income and taxable income, have been widely regarded as an important indicator because they capture not only mechanical differences arising from divergent accounting standards and tax regulations but also convey information about the structure and quality of reported earnings [1,2].

Empirical studies provide substantial evidence that BTDs are associated with earnings persistence. However, the direction of this association and the informational implications of BTDs for earnings persistence remain unsettled in the literature. Lev and Nissim [1] argue that BTDs, particularly the deferred tax component, may convey favorable information about firms’ future earnings growth and are therefore associated with higher earnings persistence. In contrast, Hanlon [2] contends that large BTDs, whether positive or negative, often reflect greater reliance on transitory earnings components and are consequently linked to lower earnings persistence.

Subsequent studies, including Blaylock et al. [3] and Jackson [4], further support Hanlon’s [2] interpretation by showing that large BTDs are associated with higher financial risk and lower predictability of future earnings. To reconcile these divergent findings, Atwood et al. [5] shift the analytical focus from the magnitude of BTDs to the degree of book–tax conformity, arguing that the persistence implications of earnings depend more critically on institutional settings than on earnings management (EM) behavior alone.

This unresolved debate is the primary motivation for the present study. For investors, auditors, and other users of financial statements, the practical issue is not only whether BTDs are associated with future earnings but also when they become more informative about the sustainability of current earnings. Interpreting BTDs in isolation may be misleading because they can arise from multiple sources, including timing differences, accounting discretion, tax planning, and institutional differences between financial reporting and tax rules. This concern is heightened during periods of sales decline, when firms face operating disruptions, working-capital adjustments, cost restructuring, and potentially larger transitory earnings components. Therefore, examining BTDs jointly with sales decline and earnings components is necessary to assess whether BTDs provide incremental information about earnings persistence under adverse operating conditions.

Building on the frameworks of Hanlon [2] and Atwood et al. [5], more recent studies show that the effect of BTDs on earnings quality is heterogeneous across firms and depends on firms’ financial characteristics and specific operating conditions [6,7]. In particular, Anderson and Rahiminejad [8] examine operating state as a conditioning factors that shape both the magnitude and direction of this association. Their findings indicate that the informational implications of BTDs for earnings persistence vary systematically with firms’ operating states and are especially pronounced during periods of sales decline.

From this perspective, even without opportunistic behavior, reactive operational adjustments during sales contractions—such as cost restructuring, working-capital realignments, or asset write-downs—can undermine the sustainability of reported earnings. As a result, earnings persistence may weaken not solely because of accounting or tax-related distortions, but also due to fundamental disruptions in the earnings-generation process under adverse operating conditions.

Although Hanlon [2] and Blaylock et al. [3] show that large BTDs are generally associated with lower earnings persistence, the implications of BTDs may differ depending on whether the differences are positive or negative and on the earnings component through which persistence is examined. Large positive BTDs may be associated with income-increasing accruals, timing differences, or tax deferral, whereas large negative BTDs may reflect conservative recognition, impairment-related adjustments, non-deductible expenses, or other institutional differences between accounting and taxable income. However, prior studies do not fully establish whether BTDs continue to provide incremental information after controlling for the fundamental components of earnings, namely operating cash flows and accruals.

Recent evidence from Anderson and Rahiminejad [8] and Le et al. [9] indicates that earnings persistence depends critically on both cash-flow and accrual components. Because these components are subject to transitory shocks, their instability can weaken the link between current and future earnings without necessarily implying opportunistic reporting. Prior studies also suggest that EM and tax avoidance (TAXAD) may be embedded in BTDs and may affect earnings persistence through different channels [2,3,8]. These arguments imply that BTDs should be examined jointly with earnings components, reporting-related behavior, and firms’ operating conditions.

However, this body of research has not systematically examined whether BTDs retain incremental information about earnings persistence when operating state and earnings components are considered together. In particular, prior studies have provided limited evidence on whether the association remains after jointly considering cash-flow disruptions, accrual components, reporting discretion, tax avoidance, and institutional accounting–tax differences. This distinction is important because different mechanisms imply different interpretations of BTDs for financial statement users. Without jointly considering sales decline, cash flows, accruals, EM, and TAXAD, the role of BTDs in predicting future earnings may be overstated, understated, or misinterpreted.

This study uses Vietnam as an institutional setting where BTDs are likely to be informative about earnings persistence for three reasons. First, financial reporting and corporate income taxation in Vietnam are governed by distinct regulatory logics. Financial statements are prepared under Vietnamese Accounting Standards and applicable accounting regulations, whereas taxable income is determined under corporate income tax rules that impose specific requirements on deductible expenses, depreciation, provisions, tax incentives, and loss carry-forwards [10,11]. Vietnam’s accounting environment has also undergone significant regulatory changes during the study period. Circular No. 200/2014/TT-BTC, which applies from the 2015 financial reporting period, represents an important reform in the preparation and presentation of corporate financial statements. These differences may create gaps between accounting and taxable income. Second, Vietnam’s corporate income tax system includes sector-, location-, and project-based incentives, meaning that the tax consequences of reported income may vary across firms with similar accounting performance [11]. Prior evidence also suggests that TAXAD is a relevant issue among Vietnamese firms [12]. These institutional features are consistent with the broader view that BTDs may reflect both reporting choices and tax-related institutional differences rather than opportunistic behavior alone [5,13]. Third, when firms experience sales declines, adjustments in working capital, provisions, impairments, and restructuring-related expenses may affect accounting and taxable income differently. Such operating states can increase the transitory components of earnings and alter the information content of BTDs for predicting future earnings [8,9]. Taken together, these features suggest that BTDs may contain incremental information about earnings persistence, particularly when firms face adverse operating conditions.

The incremental contribution of this study lies in extending the conditional BTD–earnings persistence framework in several respects. First, the study examines the framework in Vietnam, where differences between financial reporting and tax regulations, together with firm-specific tax incentives, may generate BTDs whose economic meaning differs from that observed in more developed institutional settings. Second, the analysis decomposes current earnings into operating cash flows and accruals, allowing the study to assess whether the persistence implications of BTDs and sales decline operate through different earnings components. Third, the study distinguishes between large positive and large negative BTDs and documents an asymmetric pattern in which LPBTDs provide a more stable signal of lower earnings persistence than LNBTDs. Finally, the study evaluates whether the main results remain after controlling for earnings management and tax avoidance and under alternative measurements of sales decline, sample composition, and BTD classification. Together, these extensions provide context-specific evidence on both the usefulness and the limitations of sales decline as a conditioning factor in the BTD–earnings persistence relation.

The remainder of the paper is organized as follows. Section 2 outlines the theoretical background and hypotheses. Section 3 describes the data and research design. Section 4 presents the empirical results, discussion, and robustness checks. Section 5 concludes with implications, limitations, and directions for future research.

## 2. Theoretical background and literature review

### 2.1. Theoretical background

Accounting theory and capital-market research regard earnings, particularly earnings persistence, as informative about firms’ future prospects. Earnings persistence is therefore widely regarded as an important attribute of accounting information for firm valuation and investment decisions [14–16]. From a decision-usefulness perspective, earnings are informative only to the extent that they reflect and predict future economic outcomes.

Reported earnings consist of two fundamental components: operating cash flows and accruals [2]. Operating cash flows are generally considered more persistent because they are less directly affected by accrual estimation and recognition choices, whereas accruals capture timing adjustments in revenue and expense recognition and are therefore more likely to reverse in subsequent periods [17,18]. Empirical evidence consistently shows that in models predicting future earnings, the coefficient on cash flows is typically larger than that on accruals, implying that cash flows contribute more strongly to earnings persistence [2,3,18,19]. Accordingly, a proper assessment of earnings persistence requires jointly considering both components rather than attributing changes in persistence solely to aggregate earnings or accruals.

BTDs, defined as the difference between financial accounting income and taxable income, provide an important context for examining earnings persistence. Financial accounting aims to represent firms’ economic performance and provide decision-useful information to investors, whereas the tax system focuses on determining tax liabilities under legal rules. Because the two systems differ in objectives, recognition principles, and degrees of flexibility, BTDs may arise from temporary and permanent differences, accounting choices, and tax strategies [1–3]. Prior studies show that tax-related information disclosed in financial statements can provide useful signals about the persistence and quality of earnings [1,2].

Signaling theory concerns how external users interpret an observable BTD, rather than how the difference is generated. A large BTD can alert users to potentially transitory earnings, but its sign alone does not identify whether its source is reporting discretion, legitimate tax planning, or accounting–tax rules [1,2,5,13]. Thus, the signal is informative only in conjunction with the firm’s operating state and the composition of earnings.

Agency theory concerns one possible source of the signal: information asymmetry can permit managerial discretion in accrual recognition and tax decisions [3,20–22]. Positive accounting theory specifies incentives, such as compensation, debt contracts, and political costs, under which particular accounting choices may be made [20–22]. These theories operate at different levels: signaling theory describes users’ interpretation; agency theory identifies the scope for discretion; and positive accounting theory identifies incentives for particular choices. They are complementary when discretion generates transitory differences, but institutional and mechanical BTDs need not involve managerial opportunism [5,13]. The observed association therefore cannot distinguish among these mechanisms by itself.

Sales decline provides an operating context in which the informational role of BTDs may change. When firms experience declining sales, they often face weaker demand, working-capital adjustments, cost restructuring, provisions, impairments, and other non-recurring adjustments. These changes may affect accounting income and taxable income differently, thereby increasing the transitory components of earnings and altering the information content of BTDs. In this sense, sales decline provides a common operating context for the informational and agency perspectives. It may make accounting–tax differences more salient to external users while also altering firms’ operational adjustments and, in some cases, reporting incentives under adverse conditions.

Recent studies further suggest that the informational implications of BTDs remain an active area of research. Floropoulos et al. [13] show that the association between book–tax conformity and EM varies across institutional settings, while Rahiminejad [7] documents that large BTDs are linked to financial distress and firm efficiency. More directly, Anderson and Rahiminejad [8] show that sales declines condition the relationship between BTDs and earnings persistence, and Le et al. [9] provide recent evidence that BTDs are associated with earnings quality in Vietnam. These studies motivate examining whether the BTD–earnings persistence association is jointly shaped by earnings components, operating states, and institutional context.

This conditional perspective differs from prior approaches in two respects. First, it does not assume that the persistence implications of BTDs are constant across firms or periods. Instead, it allows the association between BTDs and future earnings to vary with operating conditions. Second, it separates earnings composition from tax-related reporting signals. Cash flows and accruals capture the mechanical structure of earnings, whereas BTDs may contain additional information about accounting–tax differences, reporting choices, and institutional tax rules. Therefore, if BTDs remain associated with earnings persistence after controlling for cash flows and accruals, this suggests that BTDs convey information beyond the basic earnings decomposition.

Synthesizing these arguments, this study conceptualizes earnings persistence as a conditional attribute. BTDs are treated not as causal determinants of changes in earnings persistence but as observable indicators of firms’ reporting environments, tax-related differences, and earnings composition. Sales decline is treated as a conditioning operating state that may alter the association between BTDs and earnings persistence. Jointly examining cash-flow and accrual components allows the analysis to assess whether the observed associations are reflected in the structure of earnings, accounting–tax differences, or their interaction with adverse operating conditions.

Importantly, the direct effect of an adverse operating state should be distinguished from an incremental BTD-specific amplification effect. A decline in sales may directly weaken earnings persistence by disrupting demand, working-capital turnover, capacity utilization, and cost adjustment processes. Because costs do not necessarily adjust symmetrically or contemporaneously with changes in sales, current earnings during periods of contraction may include operating components that are less representative of future performance [8,23,24]. However, this direct operating-state effect does not necessarily imply that the association between BTDs and earnings persistence strengthens during the same period.

The hypotheses distinguish among these mechanisms. H1 examines whether BTD groups differ in earnings persistence without attributing every BTD to discretionary reporting; H2 examines the direct operating effect of a sales decline. H3 and H4 examine an additional change in the BTD–persistence association during a sales decline, separately by BTD sign. These interaction predictions require operating shocks to coincide with the underlying sources of BTDs and therefore need not follow mechanically from either H1 or H2. The empirical tests assess these conditional associations, not the distinct causal contributions of the three theoretical perspectives.

A systematic amplification effect would require the economic sources of BTDs to be related to the accounting or operational adjustments induced by declining sales. Prior research suggests that BTDs are heterogeneous and may arise from temporary and permanent differences, accounting discretion, tax planning, and institutional differences in book–tax conformity [1–5,13,20]. In the Vietnamese context, tax incentives, statutory depreciation rules, non-deductible expenses, and other accounting–tax regulatory differences may also generate BTDs without being directly related to contemporaneous changes in sales [10,11]. Accordingly, the proposed conditioning mechanism should be viewed as conditional on the economic source of BTDs rather than as an automatic consequence of an adverse operating state. This theoretical refinement provides the conceptual foundation for the hypotheses developed in the following section.

Based on the theoretical arguments above, the conceptual framework is presented in Fig 1.

**Fig 1:**
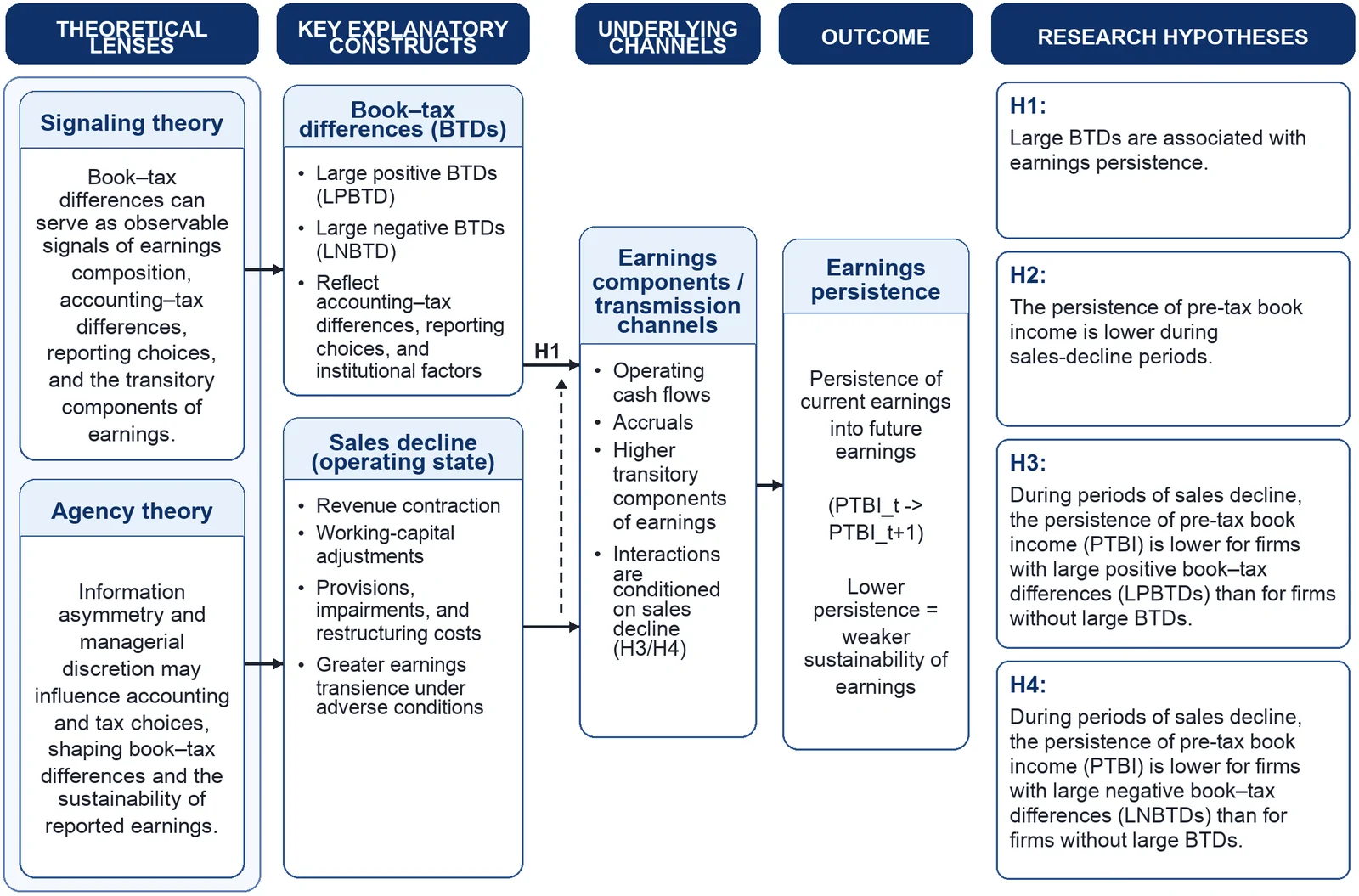
Conceptual framework.

The figure illustrates the conceptual framework of the study. Signaling theory and agency theory provide the theoretical foundation for examining the relationships among book–tax differences (BTDs), sales decline, operating cash flows, accruals, and earnings persistence. Operating cash flows and accruals represent the underlying earnings components through which earnings persistence is examined. Arrows indicate the hypothesized relationships tested in this study.

The framework links signaling and agency perspectives to BTDs and sales decline, identifies cash flows and accruals as the underlying earnings components through which persistence is examined, and positions earnings persistence as the primary outcome.

To clarify how the present study relates to prior literature, Table 1 summarizes the main research streams on BTDs, earnings persistence, operating state, and institutional settings. Prior literature provides strong evidence that BTDs are informative about future earnings, but it also suggests that their implications vary across institutional environments, firm characteristics, and earnings components. This study builds on these streams by examining whether a sales decline alters the information content of BTDs for earnings persistence in Vietnam.

**Table 1:** Summary of prior literature on BTDs, earnings persistence, and operating conditions.

Research stream	Representative studies	Main findings	Relevance to this study
BTDs and earnings persistence	Lev and Nissim [1]; Hanlon [2]; Blaylock et al. [3]; Jackson [4]	BTDs contain information about future earnings, but the direction and interpretation vary across studies. Large BTDs are often associated with lower earnings persistence.	Provides the baseline rationale for H1.
Book–tax conformity and institutional setting	Atwood et al. [5]; Floropoulos et al. [13]	The implications of BTDs and book–tax conformity depend on institutional settings and reporting incentives.	Supports the argument that Vietnam’s accounting–tax environment matters.
BTDs, financial distress, and firm conditions	Noga & Schnader [6]; Rahiminejad [7]	Large BTDs may be associated with financial distress, bankruptcy risk, or firm efficiency.	Supports the argument that the implications of large BTDs vary with firms’ financial and operating conditions.
Sales decline and operating state	Banker and Chen [23]; Banker et al. [24]; Anderson and Rahiminejad [8]	Sales decline and cost adjustments affect earnings prediction and earnings persistence.	Provides the basis for H2 and the conditioning role in H3–H4.
Earnings components	Dechow [17]; Sloan [18]; Dechow and Ge [25]	Cash flows and accruals differ in persistence; special items and transitory components reduce earnings persistence.	Supports the decomposition into PTCF and PTACC.
Emerging market and Vietnam evidence	Le et al. [9]; Ha et al. [12]	BTDs and tax-related reporting issues are relevant in Vietnam.	Justifies Vietnam as the institutional setting.

### 2.2. Research hypotheses

Before developing the hypotheses, it is important to clarify how this study defines and classifies BTDs. BTDs represent the difference between financial accounting income and taxable income and may arise from temporary timing differences, permanent differences, accounting discretion, tax planning, or institutional tax rules. Following prior research, this study distinguishes between large positive and large negative BTDs because their signs may reflect different underlying economic processes. Large positive BTDs occur when accounting income exceeds taxable income and may be associated with income-increasing accruals, timing differences, tax deferral, or preferential tax treatment. Large negative BTDs occur when taxable income exceeds accounting income and may reflect conservative accounting recognition, impairment losses, restructuring charges, non-deductible expenses, or other non-recurring reductions in accounting income.

This distinction is particularly relevant in Vietnam. Preferential corporate income tax regimes based on industry, location, and investment projects may legitimately produce positive BTDs during tax-holiday or reduced-tax periods without necessarily signaling opportunistic financial reporting [10,11]. LPBTDs may therefore reflect a more homogeneous set of accounting–tax differences. By contrast, LNBTDs may stem from a broader, more heterogeneous set of firm-specific events, including impairments, restructuring costs, conservative recognition, and non-deductible expenses [1,2,4,26]. Consequently, the persistence implications of LPBTDs and LNBTDs may vary across firms and over time, providing a theoretical basis for examining the two classifications separately in the following hypotheses.

#### 2.2.1. Book–tax differences (BTDs) and earnings persistence.

Empirical evidence from the international literature indicates that firms with large BTDs often show lower earnings persistence in subsequent periods. Hanlon [2] shows that firms with large BTDs, whether positive or negative, exhibit lower earnings persistence than firms with small BTDs. Subsequent studies extend this evidence by showing that large BTDs may reduce the predictability of future earnings through both accrual and cash-flow components, indicating a greater contribution of transitory earnings elements [2,3,18]. Accordingly, many studies employ aggregate BTD measures or classify firms by the magnitude and sign of BTDs to capture heterogeneity in the informational implications of BTDs for earnings attributes [2,6].

Although some studies report mixed evidence on the predictive power of BTDs for future earnings [27], the overall body of evidence suggests that large BTDs are informative about earnings persistence. Similar patterns have been documented in emerging markets, including Vietnam, where BTDs are associated with earnings stability and the persistence of earnings components [9].

Synthesizing these arguments, this study views BTDs as signals of earnings quality. Larger BTDs are expected to be associated with a greater share of transitory earnings components and, consequently, lower earnings persistence in subsequent periods. Based on this reasoning, the following hypothesis is proposed:

**Hypothesis H1.** Firms with large book–tax differences exhibit lower earnings persistence than firms without large book–tax differences.

#### 2.2.2. Sales decline and earnings persistence.

Research in management and financial accounting indicates that sales declines reflect disruptions in firms’ operating conditions and are often accompanied by operational responses, including capacity adjustments, production restructuring, cost reductions, changes in credit policies, increased provisioning, or asset write-downs [23,24]. These responses can increase the non-recurring components of earnings through two primary channels. First, operating cash flows become more volatile due to weakened demand and changes in working capital turnover. Second, transitory accruals tend to rise as firms rely more heavily on accounting estimates and adjustments associated with declining operations. Consistent with the literature on accrual estimation and earnings persistence, increases in transitory cash-flow and accrual components weaken the association between current and future earnings [17,18,28].

From an operations-based perspective, sales declines do not necessarily reflect changes in reporting incentives or deliberate EM but rather adaptive responses to adverse business conditions. In such settings, current earnings may be less representative of firms’ long-term earnings-generating capacity because cash-flow processes are disrupted and operational adjustments affect both costs and accounting estimates [8]. This perspective highlights the conditioning role of operating states, whereby earnings attributes—including earnings persistence—depend critically on firms’ business environments. When sales decline, both cash flows and accruals are likely to become less persistent, thereby reducing the ability of current earnings to predict future earnings [8,19,29].

Empirical evidence from emerging markets further supports the view that earnings persistence weakens during periods of operating state, particularly sales contractions. Evidence from emerging markets further indicates that earnings attributes are sensitive to operating volatility and institutional conditions. In Vietnam, recent research confirms the relevance of BTDs for earnings quality, although the role of sales decline in earnings persistence remains insufficiently examined [9].

Synthesizing these arguments, this study expects a sales decline to be negatively associated with the persistence of pre-tax book income (PTBI), reflecting the role of operating conditions in earnings sustainability. Accordingly, the following hypothesis is proposed:

**Hypothesis H2.** The persistence of pre-tax book income is lower during periods of sales decline.

H1 and H2 establish the two baseline conditions for the integrated framework. H1 predicts that BTDs are associated with lower earnings persistence because they may capture transitory earnings components, reporting discretion, or tax-related differences. H2 predicts that a sales decline weakens earnings persistence by disrupting normal operating processes and increasing earnings transience. Taken together, these arguments suggest that the persistence implications of BTDs may differ during sales-decline periods. However, because LPBTDs and LNBTDs may arise from different economic sources, any conditioning effect of sales decline may vary across BTD signs rather than operate uniformly.

#### 2.2.3. Sales decline, large positive BTDs (LPBTDs), and earnings persistence.

Prior research generally associates LPBTDs with lower earnings persistence when they reflect income-increasing accruals, tax deferral, or temporary recognition differences that reverse in subsequent periods [2,3,30]. During sales-decline periods, firms may face stronger pressure to preserve reported performance while also experiencing reversals of prior accruals, reduced investment, working-capital adjustments, impairment recognition, and restructuring activities. These adjustments can generate accrual shocks and cash-flow volatility, reducing the extent to which current earnings persist into future earnings [23,24,31–35]. More recent evidence further suggests that the persistence implications of BTDs depend on operating states and institutional contexts [5–8]. In Vietnam, where accounting–tax divergence is pronounced and firms may face substantial operating volatility, recent evidence confirms that BTDs are relevant to earnings quality, providing a basis for examining whether their persistence implications vary during sales-decline periods [9]. Taken together, these arguments suggest that, when LPBTDs reflect accounting or tax adjustments associated with deteriorating operating conditions, their negative association with earnings persistence may be stronger during sales-decline periods than during non-sales-decline periods:

**Hypothesis H3.** During periods of sales decline, the persistence of pre-tax book income is lower for firms with large positive book–tax differences than for firms without large book–tax differences.

#### 2.2.4. Sales decline, large negative BTDs (LNBTDs), and earnings persistence.

Large negative BTDs (LNBTDs) occur when accounting income is relatively low compared with taxable income. Such differences may arise from conservative accounting recognition, impairment losses, restructuring charges, non-deductible expenses, timing differences, or other non-recurring reductions in accounting income [1,2,4,26]. As with LPBTDs, the informational implications of LNBTDs depend on firms’ operating states. During sales-decline periods, firms may adopt defensive operational responses, including restructuring initiatives, workforce reductions, asset divestitures, and working-capital adjustments. These actions can depress current accounting earnings, disrupt operating cash flows, and increase the volatility of both cash-flow and accrual components [24,31,32,34]. When LNBTDs arise from accounting adjustments associated with deteriorating operating conditions, taxable income may respond differently from accounting income under divergent recognition rules, potentially increasing the divergence between the two measures. Current earnings may therefore become less representative of future earnings for LNBTD firms during sales-decline periods. Accordingly, when LNBTDs capture transitory operating or accounting adjustments associated with declining sales, earnings persistence may be further weakened relative to firms without large BTDs:

**Hypothesis H4.** During periods of sales decline, the persistence of pre-tax book income is lower for firms with large negative book–tax differences than for firms without large book–tax differences.

## 3. Research method

### 3.1. Research design and data

This study uses panel data on non-financial firms listed on the Ho Chi Minh Stock Exchange (HOSE) and the Hanoi Stock Exchange (HNX) from 2013 to 2023. The initial sample comprises 677 firms listed on HOSE and HNX as of 31 December 2023. Financial statement data were manually collected from audited annual reports, including the statements of financial position, income statements, cash flow statements, and notes to the financial statements. Information on listing status, industry classification, ticker symbols, and firm identifiers was obtained from official disclosures by HOSE and HNX, as well as from firms’ annual reports.

The data collection process involved both direct extraction and manual calculation. Pre-tax book income was extracted from the income statement. Operating cash flows were obtained from the cash flow statement. Total assets, sales revenue, and other balance sheet items were collected from the statement of financial position and relevant notes. Tax-related information, including current tax expense and information used to infer taxable income, was extracted from the income tax expense note, where available. BTDs were then calculated manually as the difference between pre-tax book income and estimated taxable income, scaled by total assets. Sales decline was coded as an indicator equal to one when annual sales growth was negative and zero otherwise.

The sample selection procedure is summarized in Table 2. We first excluded 56 financial firms, including banks, insurance companies, securities firms, and other financial institutions, because their regulatory environments, balance sheet structures, income-generating mechanisms, loan-loss provisions, capital requirements, and tax treatments differ substantially from those of non-financial firms. These differences make measures of earnings persistence and BTDs not directly comparable across financial and non-financial firms. We then excluded three firms that did not apply Circular No. 200/2014/TT-BTC from fiscal year 2015 onward, because differences in accounting regimes could reduce the comparability of financial reporting data. Twelve additional firms were excluded during outlier treatment because their financial values were extreme or inconsistent after winsorization at the 1st and 99th percentiles. Finally, 281 firms were excluded because the continuous data required to construct the main variables were unavailable. The final sample consists of 325 non-financial listed firms, corresponding to 3,575 firm-year observations over the 2013–2023 period.

**Table 2:** Sample selection counts (2013–2023).

Sample selection criteria	Number of firms	Remaining firms
Firms listed on HOSE and HNX as of 31 December 2023	677	677
Less: financial firms, including banks, insurance companies, securities firms, and other financial institutions	(56)	621
Less: firms not applying Circular No. 200/2014/TT-BTC after 2014	(3)	618
Less: firms with extreme or inconsistent financial values identified during the outlier treatment process	(12)	606
Less: firms without continuous data required to construct the main variables	(281)	325
Final sample firms		325
Firm-year observations used in the main analysis, 2013–2023		3,575

All continuous variables are winsorized at the 1st and 99th percentiles to reduce the influence of extreme values. Industry and year fixed effects are included in all adjusted regressions to control for industry-specific characteristics and common time effects. The resulting dataset is balanced at the firm level for 2013–2023, yielding 3,575 firm-year observations for the main regression analyses.

Panel data are appropriate for this study because the research question requires linking current and one-year-ahead earnings for the same firm over time. This structure allows the analysis to exploit both cross-sectional variation in BTDs across firms and time-series variation in sales conditions within firms. It also allows the inclusion of industry- and year-fixed effects to control for industry-specific characteristics and common macroeconomic shocks. Therefore, panel data provide a suitable empirical setting for examining whether the association between current and future earnings varies by BTD classification and sales-decline state.

Annual data are used instead of quarterly data for three reasons. First, constructing BTDs requires current tax expense, pre-tax book income, and information from the income tax note, which are more consistently available and more reliable in audited annual reports. Second, quarterly financial statements in Vietnam generally provide less detailed tax-related information and may not allow consistent estimation of taxable income across firms. Third, earnings persistence is examined through the relationship between current-year and one-year-ahead pre-tax book income, consistent with prior studies on BTDs and earnings persistence. Using annual data, therefore, improves comparability, reduces measurement noise, and aligns the empirical design with the availability of audited accounting and tax information.

Although the sample period includes the COVID-19 pandemic, this study does not isolate the pandemic period as a separate research setting because the objective is not to estimate the specific effect of COVID-19 on earnings persistence. Instead, the study treats sales decline as a firm-year operating state that may arise from multiple sources, including firm-specific conditions, industry shocks, macroeconomic fluctuations, and pandemic-related disruptions. Year fixed effects are included in all regressions to control for common time-specific shocks affecting listed firms in the same year, including the broad macroeconomic effects associated with the pandemic period. Accordingly, COVID-19 is treated as part of the broader time-varying economic environment rather than as a separate event-study setting. The exclusion of fiscal years 2020–2021 is therefore considered only as an additional robustness test rather than part of the primary research design.

Because the study uses repeated firm-year observations and OLS regression models, several diagnostic tests were conducted to assess the reliability of the estimates. Multicollinearity was examined using variance inflation factors (VIFs). Heteroskedasticity was assessed using the Breusch–Pagan/Cook–Weisberg and White tests. First-order serial correlation in the panel structure was examined using the Wooldridge test. These tests were conducted for the baseline PTBI model, the integrated PTBI–BTD–SD model, and the integrated earnings-component model. Because the tests indicate heteroskedasticity and within-firm serial correlation, all reported regressions use standard errors clustered at the firm level. Industry and year fixed effects are included in the adjusted specifications to control for industry-specific characteristics and common time effects.

The main adjusted specifications include industry and year indicators and firm-clustered standard errors. Industry indicators control for time-invariant differences across industries, while year indicators absorb shocks common to all firms. Because these adjustments do not eliminate unobserved firm heterogeneity or reverse causality, we conduct additional sensitivity analyses using firm and year fixed effects, a common estimation sample, lagged firm-level controls, and lagged BTD indicators. These tests mitigate concerns regarding time-invariant firm characteristics, sample comparability, and temporal ordering, but they do not identify an exogenous change in BTDs or eliminate unobserved time-varying confounding. Accordingly, all estimated interaction effects are interpreted as conditional associations rather than causal effects.

The empirical design uses a structured sequence of regression models to examine associations among BTDs, sales decline, and earnings persistence. Consistent with Hanlon [2], Blaylock et al. [3], and Anderson & Rahiminejad [8], earnings persistence is measured by how much current earnings explain future earnings, while sales decline is treated as a conditioning operating state rather than a continuous performance shock.

For transparency and ease of interpretation, the full set of regression models is summarized in Table 3, which serves as an empirical roadmap linking each specification to its corresponding hypothesis, focal coefficients, and the associated result tables. Detailed regression equations are provided in S1 Appendix to facilitate replication.

**Table 3:** Empirical models, hypotheses, and expected coefficient signs.

Model No.	Regression model	Tested hypothesis / purpose	Key coefficient(s)	Expected sign(s)	Related literature	Referenced results
(1)	Baseline model: PTBI persistence	Measures the persistence of pre-tax book income	β1 (PTBIt)	β1 > 0	Hanlon [2]; Blaylock et al. [3]; Anderson and Rahiminejad [8]	Table 8 – Panel A; Eq. (A1) in S1 Appendix
(2)	Sales-decline (SD) model	H2: Earnings persistence is lower during sales decline	β3 (SD × PTBIt)	β3 < 0	Anderson and Rahiminejad [8]	Table 9 – Model (1); Eq. (A2) in S1 Appendix
(3)	BTD model without SD	H1: Large BTDs reduce earnings persistence	β4 (LNBTD × PTBIt); β5 (LPBTD × PTBIt)	β4 < 0; β5 < 0	Hanlon [2]; Blaylock et al. [3]	Table 9 – Model (2); Eq. (A3) in S1 Appendix
(4)	Integrated SD × BTD model	H3–H4: Sales decline conditions the association between BTDs and earnings persistence	β10 (SD × LNBTD × PTBIt); β11 (SD × LPBTD × PTBIt)	β10 < 0; β11 < 0	Anderson and Rahiminejad [8]	Table 9 – Model (3); Eq. (A4) in S1 Appendix
(5)	Component-based model: PTCF and PTACC	Decomposes earnings persistence into cash flows and accruals	β1 (PTCFt); β2 (PTACCt)	β1 > β2 > 0	Sloan [18]; Hanlon [2]	Table 8 – Panel B; Eq. (A5) in S1 Appendix
(6)	SD model with earnings components	Examines whether sales decline reduces the persistence of cash flows and accruals	β4 (SD × PTCFt); β5 (SD × PTACCt)	β4 < 0; β5 < 0	Anderson and Rahiminejad [8]	Table 10 – Model (1); Eq. (A6) in S1 Appendix
(7)	BTD model with earnings components	Examines whether large BTDs are associated with the persistence of earnings components	BTD × earnings component interactions	Negative coefficients indicate lower persistence	Hanlon [2]; Anderson and Rahiminejad [8]	Table 10 – Model (2); Eq. (A7) in S1 Appendix
(8)	Integrated PTCF–PTACC–SD–BTD model	Examines whether sales decline conditions the association between BTDs and earnings-component persistence	SD × BTD × earnings component interactions	Negative coefficients indicate lower persistence	Anderson and Rahiminejad [8]	Table 10 – Model (3); Eq. (A8) in S1 Appendix
(9)	Extended model with EM	Tests whether results are sensitive to controlling for EM	Interaction terms involving EM × PTBI and EM × BTD	No directional prediction	Hanlon [2]; Blaylock et al. [3]; Anderson and Rahiminejad [8]	Table 11; Eq. (A9a) in S1 Appendix
(10)	Extended model with TAXAD	Tests whether results are sensitive to controlling for TAXAD	Interaction terms involving TAXAD × PTBI and TAXAD × BTD	No directional prediction	Hanlon [2]; Blaylock et al. [3]; Floropoulos et al. [13]	Table 12; Eq. (A9b) in S1 Appendix
(11)	Additional robustness specifications	Tests sensitivity to continuous sales-decline measurement, exclusion of 2020–2021, and annual-decile BTD classification; Test full-sample fixed effects, matched-sample fixed effects, matched-sample estimates with lagged controls, and estimates using lagged BTD indicators.	Corresponding earnings-persistence and interaction coefficients	Principal conclusions remain qualitatively consistent		Table 13 and Table 14

**Notes:** PTBI denotes pre-tax book income; PTCF denotes pre-tax operating cash flows; PTACC denotes pre-tax accruals; BTD denotes book–tax differences; LNBTD denotes large negative book–tax differences; LPBTD denotes large positive book–tax differences; SD denotes sales decline; EM denotes discretionary accruals estimated using the modified Jones model; TAXAD denotes tax avoidance indicator. Expected signs indicate the predicted direction of the interaction coefficients. Negative interaction coefficients indicate lower earnings persistence relative to the benchmark group or to non-sales-decline periods.

### 3.2. Measurement of earnings persistence

Earnings persistence is measured using a one-period autoregressive framework based on pre-tax book income. Specifically, the dependent variable is pre-tax book income in year *t + 1* (PTBI_it+1_), and the key explanatory variable is pre-tax book income in year *t* (PTBI_it_). All income variables are scaled by total assets at the beginning of year t to enhance comparability across firms and over time, consistent with standard practice in the literature [8,28]. The definitions and measurement of PTBI, PTCF, PTACC, and other variables used throughout the study are reported in Table 4.

**Table 4:** Variable definition and measurement.

Variable	Symbol	Definition and measurement	Prior literature
Pre-tax book income (t+1)	PTBI_t+1_	Pre-tax book income in year t+1, scaled by total assets at the beginning of year t.	Hanlon [2]; Blaylock et al. [3]
Pre-tax book income (t)	PTBI_t_	Pre-tax book income in year t, scaled by total assets at the beginning of year t.	Hanlon [2]; Anderson & Rahiminejad [8]
Operating cash flows	PTCF_t_	Cash flows from operating activities in year t, scaled by total assets at the beginning of year t.	Sloan [19]; Blaylock et al. [3]
Accruals	PTACC_t_	Accruals in year t, calculated as PTBI_t_ − PTCF_t_, scaled by total assets at the beginning of year t.	Dechow [18]; Sloan [19]
Book–tax differences	BTD_t_	Difference between pre-tax book income and taxable income in year t, scaled by total assets. Taxable income is estimated from current corporate income tax expense divided by the applicable statutory corporate income tax rate and should be interpreted as a proxy because taxable income is not directly disclosed in Vietnamese financial statements.	Hanlon [2]; Blaylock et al. [3]
Large positive BTD	LPBTDt	Dummy variable equal to 1 if the firm-year observation belongs to the top quintile of the BTD distribution (large positive BTD), and 0 otherwise.	Hanlon [2]; Blaylock et al. [3]
	LPBTD10	Indicator equal to one when BTD falls within the highest decile of the annual BTD distribution, and zero otherwise.	
Large negative BTD	LNBTDt	Dummy variable equal to 1 if the firm-year observation belongs to the bottom quintile of the BTD distribution (large negative BTD), and 0 otherwise.	Hanlon [2]; Noga & Schnader [6]
	LNBTD10	Indicator equal to one when BTD falls within the lowest decile of the annual BTD distribution, and zero otherwise.	
Sales decline	SD	Dummy variable equal to 1 if sales revenue in year t is lower than sales revenue in year t−1, and 0 otherwise.	Banker & Chen [24]; Anderson & Rahiminejad [8]
Sales up	SU	Dummy variable equal to 1 if sales revenue in year t is higher than sales revenue in year t−1, and 0 otherwise.	Anderson & Rahiminejad [8]
Absolute magnitude of negative annual sales growth	SDMAG	Continuous sales-decline magnitude, equal to the absolute value of negative annual sales growth and zero otherwise.	
Earnings management	EM	Discretionary accruals estimated using the modified Jones model; the absolute value is used to capture the magnitude of accrual-based EM.	Dechow et al. [18]
Tax avoidance indicator	TAXAD	TAXAD is measured using the difference between the statutory corporate income tax rate and the cash effective tax rate. The cash effective tax rate is calculated as cash taxes paid divided by pre-tax book income. Larger values indicate a greater gap between the statutory tax burden and the tax burden reflected in cash tax payments. TAXAD equals one for firm-year observations classified as tax-avoiding observations based on this measure, and zero otherwise	Hanlon [2]; Blaylock et al. [3]; Floropoulos et al. [13]

**Notes:** All continuous variables are winsorized at the 1st and 99th percentiles. Financial firms are excluded. The sample comprises non-financial listed firms in Vietnam from 2013 to 2023. Industry and year fixed effects are included in all adjusted regressions.

The baseline earnings-persistence specification is expressed as follows:


$$\begin{array}{c} {\text{PTBI}}_{\text{i},\text{t}+\text{1}}=\beta \text{0}+\beta_{\text{1}}{\text{PTBI}}_{\text{i},\text{t}}+\epsilon_{\text{i},\text{t}} \end{array}$$ (1)

The coefficient *β1* captures the persistence of pre-tax book income, with larger values indicating a stronger tendency for current earnings to carry forward into future periods. This baseline model serves as a reference for subsequent specifications that incorporate sales decline and BTDs.

### 3.3. Sales decline as a conditioning variable

Sales decline (SD) is a binary indicator equal to one when annual sales growth is negative and zero otherwise, following Anderson and Rahiminejad [8]. This binary specification captures the occurrence of an adverse operating state rather than the magnitude of sales changes. For robustness, the study also employs SDMAG, a continuous measure equal to the absolute magnitude of negative annual sales growth and zero otherwise. Larger values therefore indicate more severe sales contractions. SDMAG is used only in the additional robustness analyses reported in Section 4.8 and Table 13.

This binary measure follows Anderson and Rahiminejad [8] and is designed to capture a discrete operating state rather than the magnitude of sales changes.

To examine whether earnings persistence differs between sales-decline and non-decline periods, the following interaction specification is estimated:


$$\begin{array}{c} {\text{PTBI}}_{\text{i},\text{t}+\text{1}}=\beta \text{0}+\beta_{\text{1}}{\text{PTBI}}_{\text{i},\text{t}}+\beta_{\text{2}}{\text{SD}}_{\text{i},\text{t}}+\beta_{\text{3}}({\text{SD}}_{\text{i},\text{t}}\times {\text{PTBI}}_{\text{i},\text{t}})+\epsilon_{\text{i},\text{t}} \end{array}$$ (2)

The interaction coefficient *β3* captures the differential persistence of earnings during sales-decline periods. A negative estimate of *β3* indicates lower earnings persistence when firms experience declining sales, consistent with Hypothesis H2.

### 3.4. Book–tax differences and earnings persistence

BTDs are measured as the difference between pre-tax book income and estimated taxable income, scaled by total assets. Consistent with prior BTD studies [1,2,5], taxable income is estimated by dividing current corporate income tax expense by the applicable statutory corporate income tax rate, since taxable income is not directly disclosed in Vietnamese financial statements. This approach is widely used in the international literature when direct taxable income data are unavailable and enables comparable BTD measures across firms. However, the estimated taxable income may contain measurement error if current tax expense reflects prior-year tax adjustments, preferential tax rates, firm-specific tax incentives, or other tax-related adjustments. Accordingly, the resulting BTD measure should be interpreted as a proxy for accounting–tax differences rather than an exact measure of taxable income.

To capture heterogeneity in the informational implications of BTDs, firm-year observations are classified into three groups based on the annual distribution of BTDs: (i) LPBTD equals one if BTD falls in the top quintile of the annual BTD distribution, and zero otherwise; (ii) LNBTD equals one if BTD falls in the bottom quintile of the annual BTD distribution, and zero otherwise.

In the main analyses, LPBTD and LNBTD are defined using the highest and lowest quintiles of the annual BTD distribution, respectively. As an alternative classification, the robustness analysis uses a more stringent annual-decile threshold. LPBTD10 equals one when a firm-year observation falls within the highest 10% of the annual BTD distribution, while LNBTD10 equals one when it falls within the lowest 10%. This alternative classification tests whether the findings are sensitive to the threshold used to identify economically large book–tax differences.

Firm-year observations not classified as LPBTD or LNBTD form the benchmark group without large BTDs. Detailed definitions of LPBTD, LNBTD, and other study variables are presented in Table 4. This classification follows Hanlon [2] and Blaylock et al. [3] and allows the analysis to distinguish between large positive and large negative deviations between book and taxable income.

The association between BTDs and earnings persistence is examined by interacting PTBI_i,t_ with the LPBTD and LNBTD indicators. The interaction terms between PTBI and the BTD indicators capture whether earnings persistence differs between firms with large BTDs and the benchmark group. Negative coefficients indicate lower persistence relative to the benchmark group, while differences in sign or significance across LPBTD and LNBTD capture potential asymmetry in the information content of BTDs. The corresponding specifications are summarized in Table 3 and formally presented in S1 Appendix.

### 3.5. Integrated model: Sales decline and book–tax differences

To examine whether sales decline conditions the association between BTDs and earnings persistence, an integrated model that includes triple interactions among PTBI, BTD group indicators, and sales decline is estimated. The representative specification is:


$$\begin{array}{cc} {\text{PTBI}}_{\text{i},\text{t}+\text{1}}= & \;\beta_{\text{0}}+\beta_{\text{1}}{\text{PTBI}}_{\text{i},\text{t}}+\beta_{\text{2}}{\text{LNBTD}}_{\text{i},\text{t}}+\beta_{\text{3}}{\text{LPBTD}}_{\text{i},\text{t}}+\beta_{\text{4}}({\text{LNBTD}}_{\text{i},\text{t}}\times {\text{PTBI}}_{\text{i},\text{t}})+\beta_{\text{5}}({\text{LPBTD}}_{\text{i},\text{t}}\times {\text{PTBI}}_{\text{i},\text{t}}nonumber\\ & +\beta_{\text{6}}{\text{SD}}_{\text{i},\text{t}}+\beta_{\text{7}}({\text{SD}}_{\text{i},\text{t}}\times {\text{PTBI}}_{\text{i},\text{t}})\;+\beta_{\text{8}}({\text{LNBTD}}_{\text{i},\text{t}}\times \;{\text{SD}}_{\text{i},\text{t}})\;+\;\beta_{\text{9}}\;({\text{LPBTD}}_{\text{i},\text{t}}\;\times \;{\text{SD}}_{\text{i},\text{t}})\\ & +\;\beta_{\text{1}\text{0}}({\text{LNBTD}}_{\text{i},\text{t}}\times {\text{PTBI}}_{\text{i},\text{t}}\times {\text{SD}}_{\text{i},\text{t}})+\beta_{\text{1}\text{1}}({\text{LPBTD}}_{\text{i},\text{t}}\times {\text{PTBI}}_{\text{i},\text{t}}\times {\text{SD}}_{\text{i},\text{t}})+\epsilon_{\text{i},\text{t}} \end{array}$$ (3)

The triple-interaction coefficients *β10* and *β11* capture whether the persistence of earnings associated with large negative and large positive BTDs, respectively, differs across sales-decline and non-decline periods. Negative estimates of *β10* and *β11* are consistent with Hypotheses H4 and H3, respectively. The two-way interaction terms between SD and the BTD indicators are included to account for level differences in future earnings across BTD groups during sales-decline periods.

### 3.6. Decomposition into cash flows and accruals

To examine the channels through which earnings persistence varies, pre-tax book income is decomposed into pre-tax operating cash flows (PTCF) and pre-tax accruals (PTACC), with PTACC defined as PTBI minus PTCF. This decomposition follows Sloan [18], Hanlon [2], and Anderson & Rahiminejad [8].

Regression specifications replace current PTBI_i,t_ on the right-hand side with its two components, PTCF_i,t_ and PTACC_i,t_, while retaining PTBI_i,t+1_ as the dependent variable. Interactions between these components, sales decline, and BTD indicators are used to assess whether persistence varies across earnings components. These specifications help identify whether the observed associations are primarily reflected in operating cash flows, accruals, or both. The full set of component-level specifications is summarized in Table 3 and detailed in S1 Appendix.

### 3.7. Extensions controlling for earnings management and tax avoidance

As additional robustness checks, the baseline and integrated models are extended to control for EM and TAXAD. EM is proxied by discretionary accruals estimated using the modified Jones model. EM equals one for firm-year observations with relatively high discretionary accruals and zero otherwise.

The analysis also incorporates a proxy for tax avoidance. TAXAD is measured as the gap between the statutory corporate income tax rate and the cash effective tax rate. The cash effective tax rate is calculated as cash taxes paid divided by pre-tax book income. Larger values indicate a wider gap between the statutory tax burden and the tax burden reflected in cash tax payments. Firm-year observations are classified as tax-avoiding when this gap indicates a lower cash tax burden relative to the statutory rate.

By incorporating EM and TAXAD, along with their interactions with pre-tax book income, BTD group indicators, and sales decline, these extended specifications assess whether the documented associations among BTDs, sales decline, and earnings persistence persist after accounting for accrual-based reporting discretion and tax-related behavior. EM and tax avoidance are included primarily as control variables to mitigate potential omitted-variable bias in estimating the association between BTDs, sales decline, and earnings persistence, rather than as the primary constructs of interest. Accordingly, the results from these models are interpreted as robustness checks of the main findings rather than as causal tests of earnings management or tax-planning mechanisms.

### 3.8. Additional robustness tests

Three additional robustness tests assess whether the principal findings are sensitive to alternative variable measurement, sample composition, and BTD classification. First, the binary sales-decline indicator is replaced with SDMAG, a continuous measure of the magnitude of sales decline. The fully specified PTBI and earnings-component models are re-estimated with SDMAG and its corresponding two- and three-way interaction terms.

Second, all fully specified models are re-estimated after excluding observations from fiscal years 2020 and 2021. This subsample analysis assesses whether the baseline findings are disproportionately influenced by the unusual operating conditions associated with the COVID-19 period. The exclusion serves solely as a robustness test rather than as an event-study design intended to estimate the causal effect of the pandemic.

Third, large positive and negative BTDs are reclassified using the highest and lowest annual deciles of the BTD distribution. LPBTD10 and LNBTD10 replace the baseline quintile-based indicators in the fully specified PTBI and earnings-component models. Except for the alternative sales-decline measure, sample composition, or BTD classification, all model specifications, fixed effects, and firm-clustered standard errors remain identical to those used in the baseline analyses.

Finally, to address concerns regarding firm heterogeneity, sample comparability, and temporal ordering, we estimate additional specifications reported in Table 14. These analyses employ firm and year fixed effects with standard errors clustered by firm. We first estimate the models using the full sample and then re-estimate them on the common sample available for the lagged-control specifications. The third specification includes one-year-lagged firm size, leverage, and asset tangibility. The final specification replaces the current BTD indicators and their associated interaction terms with one-year-lagged BTD indicators. These analyses are treated as sensitivity tests rather than causal identification strategies.

The corresponding empirical results are reported in Tables 8–12, and the full regression equations are provided in S1 Appendix. The definitions, measurement procedures, and data sources for all variables used in the empirical analyses are summarized in Table 4. The variable construction follows prior studies on earnings persistence, book–tax differences, earnings management, and tax avoidance to ensure consistency and comparability with the existing literature.

## 4. Results and discussions

### 4.1. Descriptive statistics

Table 5 presents descriptive statistics for the full sample and for the two subsamples defined by sales trends (sales-up and sales-down). Overall, the distributions of pre-tax book income and its components exhibit substantial dispersion across firm-year observations, indicating considerable heterogeneity in operating conditions and earnings structures.

**Table 5:** Descriptive statistics.

Variable	Observations	Mean	SD	Min	Max
**Panel A: Full Sample**					
PTBI_i,t+1_	3,575	0.0693	0.0855	-0.1418	0.4109
PTBI_i,t_	3,575	0.07	0.087	-0.1271	0.4432
PTCF_t_	3,575	0.0643	0.1267	-0.2968	0.426
PTACC_t_	3,575	0.0059	0.1214	-0.3438	0.4019
BTD_t_	3,575	0.0032	0.0383	-0.1429	0.1411
LNBTD_t_	3,575	0.2028	0.4021	0	1
LPBTD_t_	3,575	0.1997	0.3998	0	1
EM_t_	3,575	0.2025	0.4019	0	1
TAXAD_t_	3,575	0.2017	0.4013	0	1
**Panel B: Sales-Up Sample**					
PTBI_i,t+1_	2,099	0.0847	0.0868	-0.1418	0.4109
PTBI_i,t_	2,099	0.0727	0.0864	-0.1271	0.4432
PTCF_t_	2,099	0.0689	0.1316	-0.2968	0.426
PTACC_t_	2,099	0.0041	0.1216	-0.3438	0.4019
BTD_t_	2,099	0.0078	0.0375	-0.1429	0.1411
LNBTD_t_	2,099	0.1929	0.3947	0	1
LPBTD_t_	2,099	0.233	0.4228	0	1
EM_t_	2,099	0.203	0.4023	0	1
TAXAD_t_	2,099	0.1758	0.3807	0	1
**Panel C: Sales-Down Sample**					
PTBI_i,t+1_	1,472	0.0475	0.0787	-0.1418	0.4109
PTBI_i,t_	1,472	0.0663	0.0877	-0.1271	0.4432
PTCF_t_	1,472	0.0576	0.1193	-0.2968	0.426
PTACC_t_	1,472	0.0087	0.1212	-0.3438	0.4019
BTD_t_	1,472	-0.0034	0.0385	-0.1429	0.1411
LNBTD_t_	1,472	0.2167	0.4121	0	1
LPBTD_t_	1,472	0.1515	0.3587	0	1
EM_t_	1,472	0.2018	0.4015	0	1
TAXAD_t_	1,472	0.2371	0.4254	0	1

For the full sample (Panel A), both current and future pre-tax book income (PTBI_i,t_ and PTBI_i,t+1_) have positive means, indicating that, on average, firms report positive pre-tax book income. Pre-tax operating cash flows (PTCF) are also positive on average, whereas pre-tax accruals (PTACC) are centered near zero and have a relatively large standard deviation, reflecting notable variability in the accrual component of earnings. BTDs and the EM proxy (EM) are both positive, consistent with cross-sectional differences in accounting choices, timing differences, and tax positions across firms. The effective tax rate (ETR) has a wide distribution, consistent with prior evidence documenting heterogeneity in firms’ tax outcomes.

When the sample is split by sales trends, several distributional differences emerge. Compared with the sales-up subsample, sales-down firms report lower average pre-tax book income, lower operating cash flows, a lower incidence of LPBTDs, a higher incidence of LNBTDs, and higher TAXAD values. These differences suggest that firms experiencing sales decline tend to face weaker operating performance and greater tax-related heterogeneity. At the same time, mean pre-tax book income and operating cash flows remain positive in both subsamples. Notably, the dispersion of PTBI, PTCF, and PTACC is generally larger among sales-down firms, indicating greater uncertainty and volatility in earnings generation during periods of declining revenue.

Taken together, the descriptive statistics indicate substantial variation in earnings, cash flows, accruals, BTDs, and tax-related characteristics across firm-year observations, providing sufficient cross-sectional and time-series variation for the subsequent regression analyses.

### 4.2. Correlation

Table 6 presents the Pearson correlation matrix for the main variables. The correlation matrix shows a strong positive association between current and future PTBI and the expected negative association between operating cash flows and accruals. All pairwise correlations remain below conventional thresholds, suggesting that multicollinearity is unlikely to bias the regression results.

**Table 6:** Correlation coefficient matrix.

Variable	PTBI_i,t + 1_	PTBI_i,t_	PTCF_t_	PTACC_t_	BTD_t_	EM_t_	TAXAD_t_
PTBI_i,t+1_	1.000						
PTBI_i,t_	0.7454***	1.000					
PTCF_t_	0.5102***	0.4263***	1.000				
PTACC_t_	0.0067	0.2852***	−0.7342***	1.000			
BTD_t_	0.4693***	0.2487***	0.1431***	0.0305*	1.000		
EM_t_	0.0976***	0.0743***	−0.5088***	0.5946***	0.1009***	1.000	
TAXAD_t_	−0.2758***	−0.2335***	−0.1119***	−0.0561***	0.0533***	−0.0070	1.000

**Notes**: * p < 0.10; ** p < 0.05; *** p < 0.01

### 4.3. Diagnostic tests

The diagnostic tests in Table 7 indicate that multicollinearity is not a severe concern, as the mean VIF ranges from 1.00 to 5.32 and the maximum VIF remains below the conventional threshold of 10. The Breusch–Pagan/Cook–Weisberg and White tests reject the null hypothesis of homoskedasticity across all tested models, indicating heteroskedasticity. The Wooldridge tests also reject the null hypothesis of no first-order autocorrelation, suggesting within-firm serial correlation in the panel structure. Accordingly, all reported regressions are estimated with firm-clustered standard errors, and industry and year fixed effects are included in the adjusted specifications.

**Table 7:** Diagnostic tests for OLS regression models.

Model tested	Mean VIF	Max VIF	Breusch–Pagan p-value	White test p-value	Wooldridge p-value	Standard error adjustment
Baseline PTBI model	1.00	1.00	0.0000	0.0000	0.0000	Firm-clustered SE
Integrated PTBI–BTD–SD model	3.62	5.26	0.0000	0.0000	0.0000	Firm-clustered SE
Integrated component model	5.32	8.42	0.0000	0.0000	0.0000	Firm-clustered SE

**Notes:** VIF denotes the variance inflation factor. The Breusch–Pagan/Cook–Weisberg and White tests assess heteroskedasticity. The Wooldridge test assesses first-order serial correlation in panel data. The results indicate heteroskedasticity and first-order autocorrelation across the tested models. Therefore, all reported regressions use standard errors clustered at the firm level. Industry and year fixed effects are included in the adjusted specifications.

### 4.4. Basic tests of earnings persistence

Table 8 presents baseline tests of earnings persistence, with Panel A focusing on aggregate pre-tax book income and Panel B decomposing earnings into operating cash flows and accruals. Results are reported for the full sample and for two subsamples defined by sales conditions, namely sales-up and sales-down firms. All specifications include industry and year fixed effects, and t-statistics are computed using firm-clustered standard errors.

**Table 8:** Basic tests of earnings persistence.

Panel A: Pre-Tax Book Income			
*PTBI*_*i,t+1*_ *= β*_*0*_ *+ β*_*1*_*PTBI*_*i,t*_ *+ ε*_*i,t*_			
**Variable**	**Sales-Up Firms (1)**	**Full Sample** **(2)**	**Sales-Down Firms** **(3)**
PTBI_i,t_	0.730***	0.691***	0.644***
	(29.03)	(29.76)	(18.17)
Intercept	0.040***	0.029***	0.014
	(4.73)	(5.19)	(1.48)
Industry FE	Yes	Yes	Yes
Year FE	Yes	Yes	Yes
No. of Obs.	2,099	3,575	1,472
R-squared	0.626	0.577	0.549
**Panel B: Pre-tax book income with cash flows and accruals**			
*PTBI*_*i,t+1*_ *= β*_*0*_ *+ β*_*1*_*PTCF*_*i,t*_ *+ β*_*2*_*PTACC*_*i,t*_ *+ ε*_*i,t*_			
PTCF_i,t_	0.736***	0.719***	0.684***
	(29.38)	(32.18)	(19.06)
PTACC_i,t_	0.571***	0.560***	0.550***
	(22.11)	(27.07)	(18.11)
Intercept	0.042***	0.028***	0.012
	(4.42)	(4.70)	(1.40)
Industry FE	Yes	Yes	Yes
Year FE	Yes	Yes	Yes
No. of Obs.	2,099	3,575	1,472
R-squared	0.637	0.590	0.553

**Notes:** This table reports OLS estimates with standard errors clustered at the firm level. Panel A reports the association between current pre-tax book income and one-year-ahead pre-tax book income. Panel B decomposes current pre-tax book income into operating cash flows and accruals. All regressions include industry and year fixed effects. t-statistics are reported in parentheses. ***, **, and * denote statistical significance at the 1%, 5%, and 10% levels, respectively.

In Panel A, current pre-tax book income is positively and significantly associated with future pre-tax book income across all samples. For the full sample, the estimated coefficient on PTBI_i,t_ is 0.691 (p < 0.01), indicating substantial earnings persistence. When the sample is partitioned by sales conditions, the coefficient is higher for sales-up firms (β = 0.730, p < 0.01) and lower for sales-down firms (β = 0.644, p < 0.01). The model’s explanatory power is also higher for sales-up firms (R² = 0.626) than for sales-down firms (R² = 0.549). These results indicate that earnings persistence varies across operating conditions, with lower persistence during periods of sales decline. These findings provide preliminary evidence supporting Hypothesis H2.

Panel B presents results from the earnings decomposition model, which separates current earnings into operating cash flows and accruals. In the full sample, both operating cash flows and accruals are positively and significantly associated with future pre-tax book income, with coefficients of 0.719 for PTCF_i,t_ and 0.560 for PTACCi,t (p < 0.01). Across sales conditions, both components show higher persistence among sales-up firms than among sales-down firms. Specifically, the coefficients on PTCF_i,t_ and PTACC_i,t_ are 0.736 and 0.571, respectively, for sales-up firms (p < 0.01), compared with 0.684 and 0.550 for sales-down firms (p < 0.01). Overall, the results in Panel B indicate that both cash flows and accruals exhibit weaker persistence during periods of sales decline, consistent with the pattern observed in Panel A. This pattern suggests that sales decline reduces the persistence of both earnings components even before accounting for BTDs.

### 4.5. Earnings persistence, sales decline, and book–tax differences

Table 9 reports regression results examining the moderating effects of sales decline and BTDs on earnings persistence. Column (1) presents estimates from a model that includes sales decline; column (2) examines BTD indicators; and column (3) includes sales decline, BTD measures, and higher-order interaction terms. All specifications include industry and year fixed effects, and t-statistics are based on firm-clustered standard errors.

**Table 9:** Results of estimating models with sales-down (SD) and BTDs for the persistence of pre-tax book income.

Variable	SD Only Model (1)	No SD Model (2)	Integrated Model (3)
PTBIi,t	0.738***	0.686***	0.746***
	(31.49)	(23.60)	(24.79)
LNBTD		−0.025***	−0.007
		(−6.58)	(−1.42)
LPBTD		0.050***	0.046***
		(11.56)	(9.32)
LNBTD × PTBIi,t		0.107***	0.043
		(2.63)	(0.90)
LPBTD × PTBIi,t		−0.175***	−0.140***
		(−3.90)	(−2.79)
SD	−0.023***		−0.010***
	(−7.59)		(−3.90)
SD × PTBIi,t	−0.114***		−0.127***
	(−3.28)		(−2.71)
LNBTD × SD			−0.034***
			(−5.23)
LPBTD × SD			0.002
			(0.28)
LNBTD × PTBIi,t × SD			−0.018
			(−0.22)
LPBTD × PTBIi,t × SD			−0.080
			(−1.07)
Constant	0.039***	0.025***	0.028***
	(7.21)	(3.91)	(5.20)
Industry FE	Yes	Yes	Yes
Year FE	Yes	Yes	Yes
Observations	3,575	3,575	3,575
R-squared	0.610	0.622	0.655
Root MSE	0.0536	0.0528	0.0505

In column (1), current pre-tax book income is positively and significantly associated with future pre-tax book income (β = 0.738, p < 0.01), indicating earnings persistence. In contrast, the sales decline indicator is negative and statistically significant (β = −0.023, p < 0.01), and the interaction between sales decline and current earnings is also negative and significant (β = −0.114, p < 0.01). These results suggest that earnings persistence is lower during periods of sales decline.

In column (2), the coefficient on current earnings remains positive and significant (β = 0.686, p < 0.01). The indicator for large negative BTDs is negatively associated with future earnings (β = −0.025, p < 0.01), whereas the indicator for large positive BTDs is positively associated with future earnings (β = 0.050, p < 0.01). The interaction terms reveal asymmetric persistence effects: the coefficient on LNBTD × PTBI is positive and statistically significant (β = 0.107, p < 0.01), whereas the coefficient on LPBTD × PTBI is negative and statistically significant (β = −0.175, p < 0.01).

When sales decline and BTDs, along with higher-order interactions, are jointly included in column (3), the coefficient on current earnings remains positive and significant (β = 0.746, p < 0.01). The interaction between sales decline and current earnings remains negative and statistically significant (β = −0.127, p < 0.01). The interaction between LPBTD and current earnings also remains negative and statistically significant (β = −0.140, p < 0.01), whereas the interaction between LNBTD and current earnings is no longer significant. In contrast, the triple interaction terms involving sales decline, BTDs, and current earnings are not statistically significant. Overall, the results indicate that the conditioning role of sales decline is clearly reflected in the two-way interaction with current earnings, whereas evidence that sales decline further conditions the BTD–earnings persistence relation through three-way interactions is limited in the sample.

The insignificant three-way interactions suggest that sales decline may not be a sufficiently informative conditioning variable for identifying when BTDs contain more transitory earnings components. Although sales decline increases operational uncertainty, BTDs arise from heterogeneous sources, including temporary differences, permanent tax rules, tax incentives, and normal accounting adjustments, whose effects may not necessarily become more pronounced during periods of declining sales. Consequently, while sales decline weakens earnings persistence in general, it does not appear to systematically strengthen the association between BTDs and earnings persistence. These findings suggest that the theoretical role of sales decline may require refinement.

### 4.6. Earnings persistence, sales decline, and earnings components

Table 10 reports regression results on the persistence of earnings components, accounting for the moderating effects of sales decline and BTDs. Column (1) presents a model that combines earnings components with sales decline; column (2) incorporates BTD indicators and their interactions with earnings components; and column (3) jointly includes sales decline, BTD measures, and higher-order interaction terms. All specifications include industry and year fixed effects, and t-statistics are computed using firm-clustered standard errors.

**Table 10:** Persistence of pre-tax book income with cash flows and accruals.

Variable	Model (1) SD Only	Model (2) No SD	Model (3) Integrated
PTCFi,t	0.744***	0.718***	0.754***
	(31.09)	(25.55)	(24.07)
PTACCi,t	0.576***	0.587***	0.628***
	(23.17)	(21.10)	(17.35)
LNBTD		−0.023***	−0.008
		(−6.46)	(−1.62)
LPBTD		0.049***	0.047***
		(11.74)	(9.08)
LNBTD × PTCFi,t		0.077**	0.062
		(2.06)	(1.35)
LPBTD × PTCFi,t		−0.150***	−0.139***
		(−3.69)	(−2.75)
LNBTD × PTACCi,t		0.014	−0.048
		(0.33)	(−0.88)
LPBTD × PTACCi,t		−0.204***	−0.189***
		(−4.75)	(−3.38)
SD	−0.025***		−0.012***
	(−7.79)		(−4.59)
SD × PTCFi,t	−0.077**		−0.080*
	(−2.13)		(−1.74)
SD × PTACCi,t	−0.041		−0.086*
	(−1.17)		(−1.84)
SD × LNBTD			−0.030***
			(−4.71)
SD × LPBTD			−0.000
			(−0.06)
SD × LNBTD × PTCFi,t			−0.126
			(−1.38)
SD × LPBTD × PTCFi,t			−0.040
			(−0.57)
SD × LNBTD × PTACCi,t			−0.002
			(−0.03)
SD × LPBTD × PTACCi,t			−0.015
			(−0.20)
Constant	0.039***	0.025***	0.029***
	(6.71)	(3.73)	(5.00)
Industry FE	Yes	Yes	Yes
Year FE	Yes	Yes	Yes
Observations	3,575	3,575	3,575
R-squared	0.620	0.637	0.668
Root MSE	0.0529	0.0517	0.0495

**Notes:** This table reports OLS estimates with firm-level clustered standard errors. The dependent variable is one-year-ahead pre-tax book income, PTBIi,t + 1. All regressions include industry and year fixed effects. t-statistics are reported in parentheses. ***, **, and * denote statistical significance at the 1%, 5%, and 10% levels, respectively.

In column (1), both operating cash flows and accruals are positively and significantly associated with future pre-tax book income (β = 0.744 and β = 0.576, respectively; p < 0.01). The sales decline indicator is negative and statistically significant (β = −0.025, p < 0.01). The interaction between sales decline and operating cash flows is negative and statistically significant (β = −0.077, p < 0.05), whereas the interaction between sales decline and accruals is not statistically significant. These results suggest that sales decline weakens earnings persistence primarily through the operating cash-flow component in this specification.

In column (2), the coefficients on cash flows and accruals remain positive and statistically significant (β = 0.718 and β = 0.587, respectively; p < 0.01). The indicator for large negative BTDs is negatively associated with future earnings (β = −0.023, p < 0.01), whereas the indicator for large positive BTDs is positively associated with future earnings (β = 0.049, p < 0.01). The interaction terms reveal heterogeneity across earnings components: the interaction between LNBTD and cash flows is positive and statistically significant (β = 0.077, p < 0.05), whereas the interaction between LPBTD and cash flows is negative and statistically significant (β = −0.150, p < 0.01). For accruals, only the interaction with LPBTD is negative and statistically significant (β = −0.204, p < 0.01).

When the sales decline indicator, BTD measures, and higher-order interactions are included in column (3), the coefficients on cash flows and accruals remain positive and statistically significant (β = 0.754 and β = 0.628, respectively; p < 0.01). The sales decline indicator remains negative and significant (β = −0.012, p < 0.01). The interactions between sales decline and both earnings components are negative and weakly significant at the 10% level for operating cash flows and accruals (β = −0.080 and β = −0.086, respectively). The interaction between sales decline and LNBTD is negative and statistically significant (β = −0.030, p < 0.01), whereas the interaction between sales decline and LPBTD is not statistically significant. None of the triple interaction terms involving sales decline, BTDs, and earnings components is statistically significant. Overall, the results suggest that sales decline weakens earnings persistence through earnings components, but the additional three-way evidence is limited when using firm-clustered standard errors and controlling for industry and year fixed effects.

### 4.7. Discussion

#### 4.7.1. Book–tax differences and earnings persistence (H1).

The results in Tables 9 and 10 provide partial and asymmetric evidence for Hypothesis H1. In Table 9, the interaction between LPBTD and current pre-tax book income is negative and statistically significant in both the BTD-only and integrated models, indicating lower earnings persistence among firms with large positive BTDs relative to the benchmark group. By contrast, the interaction between LNBTD and current pre-tax book income is positive in the BTD-only model and becomes statistically insignificant in the integrated model. The component-level results in Table 10 show a similar asymmetric pattern: LPBTD is negatively associated with the persistence of both cash flows and accruals, whereas the evidence for LNBTD is weaker and varies across earnings components. These findings suggest that the information content of BTDs depends on their sign and on the earnings component through which persistence is examined.

This pattern is broadly consistent with prior research showing that BTDs contain information about future earnings, but it refines the view that all large BTDs have the same persistence implications. Hanlon [2] and Blaylock et al. [3] document lower earnings persistence among firms with large BTDs, while Jackson [4] and Atwood [5] show that BTDs are informative about future earnings. The present results indicate that, in the Vietnamese setting, large positive BTDs are more consistently associated with earnings transience, whereas large negative BTDs have more heterogeneous implications. This interpretation is consistent with the view that BTDs may arise from multiple sources, including accounting choices, tax rules, and institutional differences, and therefore, their implications for earnings persistence may vary across operating conditions.

#### 4.7.2. Sales decline and earnings persistence (H2).

The baseline interaction results in Tables 9 and 10 support Hypothesis H2, showing that earnings persistence is lower during periods of sales decline. In Table 9, the interaction between sales decline and current pre-tax book income is negative and statistically significant, indicating that contemporaneous earnings are less representative of future earnings when firms experience declining sales. Table 10 further shows that sales decline weakens the persistence of earnings components, with stronger evidence for operating cash flows than for accruals when using firm-clustered standard errors. This evidence is consistent with an operations-based perspective, under which declining sales trigger cost adjustments, working-capital changes, cash-flow disruptions, and non-recurring items that reduce earnings persistence [8,19,23,24]. These results provide the basis for examining whether sales decline changes the information content of BTDs through interaction and component-level evidence, rather than inferring the role of BTDs solely from the main effect of sales decline.

#### 4.7.3. Sales decline as a conditioning factor for BTD effects (H3 and H4).

The integrated models in Tables 9 and 10 offer useful evidence for evaluating the theoretical mechanism underlying Hypotheses H3 and H4. Although the two-way interaction between sales decline and current earnings is consistently negative and statistically significant, the three-way interaction terms involving sales decline, BTD indicators, and current earnings are not statistically significant in Table 9. These results indicate that while sales decline consistently weakens earnings persistence, it does not necessarily strengthen the information content of BTDs. In other words, the evidence supports a direct operating-state effect of sales decline but not an additional BTD-specific amplification effect. Consequently, rather than supporting the proposed three-way mechanism, the findings suggest that the theoretical explanation underlying H3 and H4 requires further refinement.

The component-level results in Table 10 yield a similar conclusion. Sales decline is negatively associated with the persistence of both cash-flow and accrual components in the integrated model, though the interaction terms are only weakly significant at the 10% level. LPBTD remains negatively associated with the persistence of both cash flows and accruals, suggesting that large positive BTDs are more consistently linked to transitory earnings components.

An additional implication of these findings concerns the asymmetry between LPBTDs and LNBTDs. LPBTDs show a more stable negative association with earnings persistence across specifications, whereas evidence for LNBTDs is considerably less consistent. This asymmetry is theoretically meaningful because the two BTD classifications are unlikely to reflect the same underlying economic processes. In the Vietnamese institutional setting, preferential corporate income tax regimes [10,11] may generate LPBTDs even when firms do not engage in opportunistic reporting. Firms benefiting from tax holidays or reduced tax rates may legitimately report accounting income that exceeds taxable income, increasing the likelihood of observing positive book–tax differences that primarily reflect institutional tax provisions rather than opportunistic financial reporting. Consequently, LPBTDs may reflect a more homogeneous institutional reporting environment, making them a relatively more stable indicator of accounting–tax divergence. By contrast, LNBTDs may arise from a much broader range of economic events, including impairment losses, restructuring costs, conservative accounting recognition, non-deductible expenses, temporary losses, and other firm-specific adjustments [1,2,4,26]. Because these sources differ substantially across firms and years, LNBTDs are likely to exhibit greater economic heterogeneity, reducing the stability of their association with future earnings persistence. However, none of the three-way interaction terms involving sales decline, BTD indicators, and earnings components is statistically significant when using firm-clustered standard errors and controlling for industry and year fixed effects. Therefore, the component-level evidence does not support a broad three-way amplification effect.

These findings refine rather than contradict prior BTD research. Hanlon [2] shows that firms with large positive or negative BTDs generally exhibit lower earnings persistence, while Blaylock et al. [3] further document that large positive temporary BTDs are associated with lower persistence when linked to TAXAD. Atwood et al. [5] also emphasize that the persistence implications of earnings depend on the degree of book–tax conformity and institutional settings. The present findings are consistent with this broader literature, showing that BTDs contain information about earnings persistence. However, they also suggest that when sales decline is introduced as an operating-state condition, the BTD–persistence relation does not necessarily intensify uniformly across all large-BTD firms.

This distinction is theoretically important. As discussed in Section 2.1, a decline in sales may directly weaken earnings persistence by disrupting firms’ operating activities, working-capital management, capacity utilization, and cost adjustment processes [8,23,24]. However, such operating disruptions do not necessarily imply that BTDs become more informative about future earnings. An incremental amplification effect would be expected only when the underlying sources of BTDs are systematically associated with the accounting or tax adjustments induced by declining sales. Prior studies indicate that BTDs are economically heterogeneous and may arise from temporary differences, permanent differences, earnings management, tax-planning activities, and institutional characteristics of the tax system [1–5,13,20]. In the Vietnamese context, BTDs may also reflect tax incentives, statutory depreciation rules, limitations on deductible expenses, and other regulatory differences between accounting and tax reporting [10,11]. These institutionally generated BTDs are not necessarily linked to contemporaneous changes in sales.

The seemingly insignificant three-way interactions may also reflect that sales declines weaken earnings persistence primarily through operating adjustments that are not fully captured by aggregate BTD indicators. Revenue contractions can disrupt cash flows, working-capital management, cost structures, and impairment recognition, thereby reducing earnings persistence regardless of whether firms report LPBTDs or LNBTDs [8,23–25].

Therefore, the absence of significant three-way interactions should not be taken as evidence against the informational role of BTDs. Rather, it suggests that the conditioning effect of sales decline depends on the underlying economic characteristics of the observed BTDs. When BTDs primarily reflect operating adjustments associated with deteriorating business conditions, sales decline could enhance their information content. Conversely, when BTDs mainly originate from institutional or tax-policy differences, sales decline and BTDs are likely to influence earnings persistence through separate rather than multiplicative channels. Accordingly, our findings refine rather than reject the original theoretical framework. The more robust evidence for LPBTDs than for LNBTDs further suggests that the informational implications of BTDs depend not only on their magnitude but also on their underlying economic source, consistent with the broader institutional perspective of Atwood et al. [5] and Floropoulos et al. [13]. This distinction appears particularly relevant in Vietnam, where preferential tax regimes may systematically generate LPBTDs without implying opportunistic reporting behavior. Taken together, these findings indicate that sales decline is an important determinant of earnings persistence, whereas its ability to strengthen the BTD–earnings persistence relation depends on the underlying economic nature of the observed BTDs. Consequently, H3 and H4 are not supported in the main analyses.

### 4.8. Robustness

In Table 11, the interaction between EM and current earnings is negative and statistically significant in the baseline and BTD-interaction specifications, suggesting that accrual-based reporting discretion is associated with weaker earnings persistence. However, this interaction becomes statistically insignificant in the fully specified sales-decline model. In Table 12, the interaction between TAXAD and current earnings remains negative, suggesting that TAXAD is associated with weaker earnings persistence. Across both robustness tests, the interaction between sales decline and current earnings remains negative and statistically significant, while the triple interactions involving sales decline, BTDs, and current earnings remain statistically insignificant. These results support the main inference that earnings persistence is lower during sales-decline periods and reinforce the conclusion that evidence for a broad three-way BTD–sales-decline mechanism is limited.

**Table 11:** Robustness checks: earnings persistence controlling for earnings management.

Variable	Baseline (1)	BTD interactions (2)	Sales decline (3)
PTBIi,t	0.723***	0.767***	0.754***
	(32.62)	(23.49)	(22.38)
EM	0.022***	0.009***	0.004
	(5.64)	(3.11)	(1.39)
EM × PTBIi,t	−0.147***	−0.115***	−0.047
	(−4.04)	(−3.63)	(−0.96)
LNBTD		−0.009*	−0.012**
		(−1.88)	(−2.48)
LPBTD		0.039***	0.037***
		(8.12)	(6.95)
LNBTD × PTBIi,t		0.046	0.088*
		(0.95)	(1.81)
LPBTD × PTBIi,t		−0.126**	−0.106*
		(−2.58)	(−1.92)
EM × LNBTD		0.012	0.026**
		(1.50)	(2.37)
EM × LPBTD		0.024***	0.031***
		(3.52)	(3.41)
EM × LNBTD × PTBIi,t			−0.185**
			(−2.09)
EM × LPBTD × PTBIi,t			−0.090
			(−1.13)
SD		−0.010***	−0.010***
		(−3.93)	(−3.89)
SD × PTBIi,t		−0.126***	−0.127***
		(−2.77)	(−2.75)
SD × LNBTD		−0.033***	−0.032***
		(−5.14)	(−5.02)
SD × LPBTD		0.000	0.000
		(0.06)	(0.03)
SD × LNBTD × PTBIi,t		−0.038	−0.047
		(−0.47)	(−0.60)
SD × LPBTD × PTBIi,t		−0.075	−0.071
		(−1.01)	(−0.96)
Constant	0.025***	0.026***	0.028***
	(4.50)	(4.74)	(4.95)
Industry FE	Yes	Yes	Yes
Year FE	Yes	Yes	Yes
Observations	3,575	3,575	3,575
R-squared	0.584	0.660	0.661
Root MSE	0.0554	0.0501	0.0501

**Notes:** This table reports OLS estimates with firm-level clustered standard errors. The dependent variable is one-year-ahead pre-tax book income, PTBIi,t+1. All regressions include industry and year fixed effects. t-statistics are reported in parentheses. ***, **, and * denote statistical significance at the 1%, 5%, and 10% levels, respectively.

**Table 12:** Robustness tests: controlling for tax avoidance.

Variable	Baseline (1)	BTD interactions (2)	Sales decline (3)
PTBIi,t	0.676***	0.687***	0.748***
	(25.10)	(22.41)	(23.69)
TAXAD	−0.023***	−0.009***	−0.007***
	(−7.03)	(−3.53)	(−3.00)
TAXAD × PTBIi,t	−0.104*	−0.287***	−0.273***
	(−1.74)	(−4.69)	(−5.51)
LNBTD		0.015***	0.016***
		(4.14)	(3.55)
LPBTD		0.045***	0.041***
		(6.99)	(6.70)
LNBTD × PTBIi,t		−0.069*	−0.072
		(−1.84)	(−1.62)
LPBTD × PTBIi,t		−0.094*	−0.051
		(−1.88)	(−1.09)
TAXAD × LNBTD		−0.094***	−0.091***
		(−14.30)	(−14.23)
TAXAD × LPBTD		0.017**	0.016**
		(2.37)	(2.32)
SD			−0.010***
			(−3.89)
SD × PTBIi,t			−0.126***
			(−2.73)
SD × LNBTD			−0.003
			(−0.58)
SD × LPBTD			0.005
			(0.60)
SD × LNBTD × PTBIi,t			−0.090
			(−1.28)
SD × LPBTD × PTBIi,t			−0.115
			(−1.47)
Constant	0.038***	0.025***	0.029***
	(6.16)	(5.16)	(6.60)
Industry FE	Yes	Yes	Yes
Year FE	Yes	Yes	Yes
Observations	3,575	3,575	3,575
R-squared	0.593	0.687	0.709
Root MSE	0.0547	0.0480	0.0463

**Notes:** This table reports OLS estimates with firm-level clustered standard errors. The dependent variable is one-year-ahead pre-tax book income, PTBIi,t+1. All regressions include industry and year fixed effects. t-statistics are reported in parentheses. ***, **, and * denote statistical significance at the 1%, 5%, and 10% levels, respectively.

Table 11 presents robustness checks that control for EM in the analysis of earnings persistence. All specifications include industry and year fixed effects, and t-statistics are based on firm-clustered standard errors. In column (1), the interaction between EM and current earnings is negative and statistically significant, indicating that accrual-based reporting discretion is associated with weaker earnings persistence in the baseline specification. In column (2), after adding BTD interactions and sales-decline terms, EM × PTBI remains negative and statistically significant. However, in column (3), which includes higher-order interactions involving EM and BTDs, EM × PTBI becomes statistically insignificant. Importantly, SD × PTBI remains negative and statistically significant, and the triple interactions involving SD, BTDs, and PTBI remain statistically insignificant. These results suggest that the sales-decline effect is not fully explained by EM, while the evidence for a three-way BTD–sales-decline mechanism remains limited.

Table 12 presents robustness tests that control for TAXAD. All specifications include industry and year fixed effects, and t-statistics are based on firm-clustered standard errors. In column (1), TAXAD is negatively associated with future earnings, and the interaction between TAXAD and current earnings is negative and weakly significant. In columns (2) and (3), TAXAD × PTBI turns negative and statistically significant, suggesting that TAXAD is associated with weaker earnings persistence after controlling for BTD indicators and sales-decline effects. The interaction between sales decline and current earnings remains negative and statistically significant in the fully specified model. However, the triple interaction terms involving sales decline, BTDs, and current earnings remain statistically insignificant. Overall, these results suggest that the main inference about lower earnings persistence during sales-decline periods is robust to controlling for TAXAD, whereas evidence for a broad three-way BTD–sales-decline mechanism remains limited.

By incorporating proxies for EM and TAXAD, these additional tests assess whether the association between BTDs and earnings persistence persists after accounting for financial reporting discretion and tax-related behavior. These results should be interpreted as additional robustness evidence rather than as evidence identifying the causal effects of reporting discretion or tax-planning behavior [2,13,36].

To further assess the stability of the baseline results, Table 13 presents three additional robustness tests using alternative variable definitions, sample composition, and BTD classification. Panel A replaces the binary sales-decline indicator with a continuous measure of sales decline (SDMAG). The estimated coefficients remain qualitatively consistent with the baseline models, indicating that the negative association between sales decline and earnings persistence is robust to an alternative measure of operating deterioration. Panel B re-estimates all models after excluding observations from the COVID-19 period (2020–2021). The principal coefficients retain their expected signs and statistical significance, suggesting that the baseline findings are not driven by pandemic-related economic conditions. Panel C adopts a more stringent classification of large book–tax differences by defining LPBTD10 and LNBTD10 as firms in the highest and lowest annual deciles of the BTD distribution, respectively. Although a small number of interaction terms show minor changes in statistical significance, the overall pattern of results remains highly consistent with the baseline analyses.

**Table 13:** Summary of additional robustness tests.

Variables	Panel A Continuous sales-decline magnitude (SDMAG)		Panel B Excluding COVID-19 (2020–2021)		Panel C Alternative BTD classification (annual deciles)	
	PTBI model	Cash flow and accrual model	PTBI model	Cash flow and accrual model	PTBI model	Cash flow and accrual model
**Current earnings persistence**						
PTBI	0.746***	–	0.736***	–	0.740***	–
PTCF	–	0.766***	–	0.751***	–	0.758***
PTACC	–	0.634***	–	0.611***	–	0.624***
**BTD effects**						
LPBTD (LPBTD10 in Panel C)	0.048***	0.048***	0.041***	0.044***	0.071***	0.073***
LNBTD (LNBTD10 in Panel C)	−0.017***	−0.016***	−0.004	−0.005	−0.010	−0.013*
LPBTD × earnings	−0.183***	PTCF=−0.165*** PTACC=−0.223***	−0.112**	PTCF=−0.132** PTACC= −0.166***	−0.208***	PTCF= −0.237*** PTACC= −0.237***
LNBTD × earnings	0.078*	PTCF = 0.069* PTACC= −0.030	0.032	PTCF = 0.050 PTACC= −0.056	0.038	PTCF = 0.073 PTACC= −0.096
**Sales-decline effect**						
Sales decline × earnings	SDMAG × PTBI = −0.822***	SDMAG × PTCF = −0.700*** SDMAG × PTACC = −0.621***	SD × PTBI = −0.117**	SD × PTCF = −0.079 SD × PTACC = −0.067	SD × PTBI = −0.150***	SD × PTCF = −0.115*** SD × PTACC = −0.120***
**Three-way interactions**	All insignificant	Only SDMAG × LPBTD × PTACC is significant at the 10% level	All insignificant	All insignificant	All insignificant	Only SD × LNBTD10 × PTCF remained significant (5%)
**Model statistics**						
Industry fixed effects	Yes	Yes	Yes	Yes	Yes	Yes
Year fixed effects	Yes	Yes	Yes	Yes	Yes	Yes
Firm-clustered standard errors	Yes	Yes	Yes	Yes	Yes	Yes
Observations	3,575	3,575	2,925	2,925	3,575	3,575
R²	0.657	0.672	0.657	0.673	0.673	0.687

**Notes:** Panel A uses a continuous measure of sales decline (SDMAG) instead of the binary indicator. Panel B excludes fiscal years 2020–2021. Panel C defines LPBTD10 and LNBTD10 as observations in the highest and lowest annual BTD deciles, respectively, replacing the baseline annual quintile classification. Only the fully specified models (Model 3) are reported. Industry and year fixed effects are included, and standard errors are clustered at the firm level. *, **, and *** indicate statistical significance at the 10%, 5%, and 1% levels, respectively.

Across all three robustness tests, the principal conclusions remain qualitatively unchanged. First, sales decline continues to reduce earnings persistence under alternative empirical specifications, confirming that this result is not sensitive to how sales decline is measured or to sample composition. Second, LPBTDs consistently show a stronger, more stable association with lower earnings persistence than LNBTDs, reinforcing the interpretation that positive and negative book–tax differences capture distinct underlying economic mechanisms in the Vietnamese institutional setting. Third, the hypothesized three-way interaction among sales decline, BTDs, and current earnings remains generally unsupported across all robustness specifications. This evidence is consistent with the refined theoretical argument developed in Section 2.1 and discussed in Section 4.7.3, namely that sales decline directly weakens earnings persistence but does not automatically strengthen the informational role of BTDs. Instead, any amplification effect depends on the underlying economic source of the observed BTDs. Overall, these additional analyses provide further evidence that the baseline conclusions are robust to alternative measures of sales decline, sample composition, and the definition of large book–tax differences.

Table 14 presents additional analyses addressing firm heterogeneity, sample comparability, and temporal ordering. Across the firm fixed-effects, matched-sample, lagged-control, and lagged-BTD specifications, the general conclusions regarding H3 and H4 remain unchanged. In Panel A, none of the three-way interactions is individually significant, and the corresponding joint Wald tests are also insignificant across all four specifications. In Panel B, only the interaction among sales decline, lagged LNBTD, and accruals is significant. However, the four lagged three-way interactions are jointly insignificant (*p* = 0.180). This isolated coefficient therefore does not provide consistent support for H3 or H4. Although these analyses mitigate concerns about omitted time-invariant firm characteristics and strengthen temporal ordering, they do not eliminate reverse causality or unobserved time-varying confounding.

**Table 14:** Robustness analyses addressing firm heterogeneity, sample comparability, and temporal ordering.

Panel A. Aggregate PTBI specification				
**Variables**	**(1) Full-sample FE**	**(2) Matched-sample FE**	**(3) Matched sample + controls**	**(4) Lagged BTD**
$PTBI_{t}$	0.412***	0.455***	0.469***	0.520***
	(0.035)	(0.042)	(0.040)	(0.042)
$LNBTD_{t}\times PTBI_{t}$	0.080**	0.096**	0.096**	
	(0.040)	(0.043)	(0.043)	
$LPBTD_{t}\times PTBI_{t}$	−0.092*	−0.111*	−0.102*	
	(0.053)	(0.057)	(0.055)	
$LNBTD_{t-\text{1}}\times PTBI_{t}$				−0.108***
				(0.038)
$LPBTD_{t-\text{1}}\times PTBI_{t}$				−0.041
				(0.071)
$SD_{t}\times PTBI_{t}$	−0.123***	−0.127***	−0.119***	−0.105**
	(0.038)	(0.042)	(0.040)	(0.044)
$SD\times LNBTD_{t}\times PTBI_{t}$	0.054	0.018	0.025	
	(0.068)	(0.076)	(0.076)	
$SD\times LPBTD_{t}\times PTBI_{t}$	−0.017	−0.028	−0.035	
	(0.072)	(0.080)	(0.080)	
$SD\times LNBTD_{t-\text{1}}\times PTBI_{t}$				0.100
				(0.067)
$SD\times LPBTD_{t-\text{1}}\times PTBI_{t}$				−0.012
				(0.109)
Joint-test p-value	0.688	0.910	0.846	0.280
Lagged controls	No	No	Yes	Yes
Lagged BTD	No	No	No	Yes
Firm and year FE	Yes	Yes	Yes	Yes
Firm-clustered SE	Yes	Yes	Yes	Yes
Observations	3,575	3,250	3,250	3,250
Firms	325	325	325	325
Within $R^{\text{2}}$	0.313	0.339	0.358	0.274
**Panel B. Three-way interactions in the component specification**				
**Three-way interactions**	**(1) Full-sample FE**	**(2) Matched-sample FE**	**(3) Matched sample + controls**	**(4) Lagged BTD**
$SD\times LNBTD_{t}\times PTCF_{t}$	−0.009	−0.048	−0.046	
	(0.071)	(0.077)	(0.077)	
$SD\times LPBTD_{t}\times PTCF_{t}$	0.009	−0.007	−0.018	
	(0.071)	(0.081)	(0.082)	
$SD\times LNBTD_{t}\times PTACC_{t}$	0.071	−0.001	−0.001	
	(0.073)	(0.079)	(0.079)	
$SD\times LPBTD_{t}\times PTACC_{t}$	0.070	0.058	0.053	
	(0.073)	(0.084)	(0.085)	
$SD\times LNBTD_{t-\text{1}}\times PTCF_{t}$				0.051
				(0.067)
$SD\times LPBTD_{t-\text{1}}\times PTCF_{t}$				0.030
				(0.098)
$SD\times LNBTD_{t-\text{1}}\times PTACC_{t}$				0.149**
				(0.067)
$SD\times LPBTD_{t-\text{1}}\times PTACC_{t}$				0.027
				(0.097)
Joint-test p-value	0.606	0.811	0.792	0.180
Observations	3,575	3,250	3,250	3,250
Firms	325	325	325	325
Within $R^{\text{2}}$	0.336	0.354	0.370	0.286

**Notes:** The dependent variable is pre-tax book income in year t+1 ($PTBI_{t+\text{1}}$). Panel A presents the aggregate earnings specification, while Panel B decomposes current earnings into pre-tax cash flows ($PTCF_{t}$) and accruals ($PTACC_{t}$). LNBTD and LPBTD indicate large negative and large positive book–tax differences; SD indicates a sales decline. Columns (1)–(4) report, respectively, full-sample fixed effects, matched-sample fixed effects, matched-sample estimates with lagged controls, and estimates using lagged BTD indicators. All models include firm and year fixed effects and the corresponding lower-order terms. Standard errors are clustered by firm and reported in parentheses. The joint-test p-value is based on a Wald test of the reported three-way interactions. *p<0.10, **p<0.05, ***p<0.01.

## 5. Conclusions, limitations, and future research

### 5.1. Conclusions and implications

This study examines the association between book–tax differences (BTDs) and earnings persistence, with particular attention to the conditioning role of sales decline, using evidence from non-financial listed firms in Vietnam. The results show that pre-tax book income is generally persistent, though persistence varies across earnings components, BTD classifications, and operating conditions. Sales decline is consistently associated with a weaker link between current and future earnings. The evidence for BTDs is asymmetric: large positive BTDs are more consistently associated with lower earnings persistence, whereas the evidence for large negative BTDs varies across model specifications and earnings components. After using firm-clustered standard errors and controlling for industry and year fixed effects, the three-way interactions among sales decline, BTD classifications, and current earnings are not statistically significant in the main analyses. Thus, the evidence supports a conditional interpretation of earnings persistence but does not support a broad three-way BTD-specific amplification mechanism.

Theoretically, the study contributes to accounting research by conceptualizing earnings persistence as a conditional attribute rather than a fixed property of reported earnings. The findings suggest that BTDs should not be interpreted solely as proxies for earnings management or tax avoidance. Instead, BTDs may serve as context-dependent indicators of accounting–tax divergence, reporting choices, institutional tax rules, and transitory earnings components. By integrating the BTD literature with an operations-based perspective on sales decline, the study extends prior research on earnings quality and tax-related accounting information. The results indicate that the persistence implications of BTDs depend on their magnitude, sign, underlying economic source, and the earnings component through which persistence is examined. At the same time, the absence of consistent three-way interaction effects suggests that sales decline directly weakens earnings persistence but does not automatically strengthen the informational role of BTDs.

The findings also have practical and policy implications. For auditors, large BTDs may signal the need for further assessment of provisions, impairments, revenue recognition, depreciation, deductible expenses, and other accounting–tax differences. Sales decline may separately signal heightened operating uncertainty, working-capital disruption, and reduced earnings sustainability. For financial analysts and investors, the results suggest that BTDs should be evaluated alongside sales trends and earnings components rather than treated as standalone indicators of earnings quality. Large positive BTDs and sales decline may each warrant closer scrutiny because both are associated with lower earnings persistence; however, the results do not indicate that their joint occurrence necessarily produces an additional amplification effect. For tax authorities and regulators, repeated or unusually large BTDs may provide useful screening information for analytical review, but they should not be interpreted as direct evidence of aggressive reporting or tax non-compliance. Such indicators should be evaluated together with firm-specific circumstances, applicable tax incentives, industry conditions, and detailed accounting and tax disclosures.

### 5.2. Limitations

This study has several limitations. First, taxable income is estimated from current corporate income tax expense because it is not directly disclosed in Vietnamese financial statements; preferential rates, tax incentives, and prior-year adjustments may introduce measurement error. Second, the earnings-management and tax-avoidance proxies do not fully capture reporting or tax strategies, and firm-level tax-incentive status is not consistently disclosed. Third, the sample requires continuous observations and is restricted to listed non-financial firms, limiting generalizability. Finally, although the additional analyses employ firm and year fixed effects, a common estimation sample, lagged controls, and lagged BTD indicators, they do not fully eliminate reverse causality or unobserved time-varying confounding. Firm fixed effects absorb time-invariant firm characteristics, whereas lagged variables improve temporal ordering; neither approach provides exogenous variation in BTDs. Firms may still adjust their reporting or tax positions in anticipation of future operating performance. The results should therefore be interpreted as conditional associations rather than causal effects.

### 5.3. Future research

Future research could use tax-return data, administrative records, or more detailed firm-level tax disclosures to validate taxable-income estimates and identify the underlying sources of BTDs. Stronger causal identification could be obtained from credible instruments, regulatory changes, tax-policy discontinuities, or other quasi-experimental settings. Future studies could also incorporate richer time-varying controls, alternative measures of earnings management and tax avoidance, longer panels, and cross-country samples.

## Supporting information

S1 Appendix Complete regression equations for the baseline earnings-persistence models, sales-decline models, book–tax difference models, earnings-component models, and extensions controlling for earnings management and tax avoidance. (DOCX)

S1 Data Firm-level financial and corporate governance data. Excel dataset containing firm identifiers, reporting years, financial and corporate governance variables, and measures used in the analyses of earnings persistence, sales declines, book–tax differences, earnings components, earnings management, and tax avoidance. (XLSX)

S2 Data Stata dataset for the regression analyses. Dataset containing firm-level observations, analysis variables, interaction terms, and estimation-sample indicators for the earnings-persistence models and their extensions. (DTA)
