# Supplementary material for: When do book–tax differences matter for earnings persistence? The role of sales decline
Source: PLoS One. 2026 Oct 28;21(10):e0344270. doi: 10.1371/journal.pone.0344270 (PMC13716203; doi:10.1371/journal.pone.0344270)
Supplement: Supplementary file 1 [file pone.0344270.s006.pdf]

Response to Reviewers
Manuscript ID: PONE-D-26-07956

Title: When Do Book–Tax Differences Matter for Earnings Persistence? The Role of Sales Decline

Dear Academic Editor and Reviewers,

We sincerely thank the Academic Editor and the three reviewers for their constructive and detailed comments on our manuscript. We have carefully revised the manuscript to address all comments. The revision substantially strengthens the motivation, theoretical framework, institutional justification, sample selection procedure, research design, diagnostic testing, interpretation of results, practical implications, limitations, and PLOS ONE formatting. We also re-estimated the main regression models using OLS with firm-clustered standard errors and industry and year fixed effects, and we revised the conclusions to avoid overclaiming beyond the evidence.

All changes have been incorporated into the revised manuscript and highlighted in the tracked-changes version. Below, we provide a point-by-point response.

1. Response to Academic Editor

Editor's comment	Response	Revisions made and location in revised manuscript
The paper requires thorough revision. All reviewers' comments must be fully addressed. The introduction needs to be more detailed and precise. The study's limitations must be described in greater detail. The practical implications and future research need to be outlined in greater detail.	We thank the Academic Editor for this guidance. We have thoroughly revised the manuscript. The Introduction now provides a clearer motivation, explains why Vietnam is an appropriate institutional setting, clarifies the conditional perspective adopted in the study, and avoids broad generalization beyond the Vietnamese setting. The Conclusions, limitations, and future research section has also been substantially expanded to clarify theoretical contributions, practical implications, limitations, and future research directions.	Introduction; Theoretical background and literature review; Research Method; Results and discussions; Conclusions, limitations, and future research.
Please ensure that the manuscript meets PLOS ONE style requirements, including referencing format.	We revised the manuscript to comply more closely with PLOS ONE formatting requirements. The reference list has been checked and reformatted using numerical citation	References; in-text citations throughout the manuscript.

	style. We corrected prior citation errors and removed "Error! Reference source not found" messages.	
For studies involving third-party data, please provide a Data Availability Statement with sufficient information.	We clarified that the data are derived from publicly available annual financial statements and corporate reports of listed firms from HOSE, HNX, and firms' official disclosures. We also prepared the dataset and Stata code for reproducibility.	Data availability statement. Before final submission, we will ensure that the dataset and Stata codes are either provided as Supporting Information or that all access information to public data sources is fully specified in accordance with PLOS ONE's data policy.
Ethics statement should appear only in the Methods section.	We revised the ethics statement to clarify that the study uses secondary publicly available corporate financial statement data and does not involve human participants, personal data, confidential information, animal subjects, or field research.	Ethics statement in the manuscript.
References and bibliography contain discrepancies, including missing reference, repeated reference, DOI issues, and "Error! Reference source not found."	We carefully checked the reference list and in-text citations. We removed citation errors, corrected the repeated reference issue, added missing references, and updated the DOI information where available. We also added recent studies from 2024 onward, including Floropoulos et al. (2024), Rahiminejad (2024), Anderson and Rahiminejad (2025), and Le et al. (2025).	References; Introduction; Theoretical background; Discussion.

2. Response to Reviewer 1

Reviewer 1 comment	Response	Revisions made and location in revised manuscript
1. The Introduction should explain why Vietnam is significant for accounting–tax separation and revenue volatility. The manuscript should explicitly argue why the Vietnamese regulatory framework	Thank you for this important comment. We have substantially revised the Introduction to explain why Vietnam is an appropriate institutional setting. We now explain that financial reporting and corporate	Introduction: paragraph on Vietnam institutional setting. Research Method: Research design and data; Table 2 Sample selection counts.

makes BTD a particularly informative signal during sales declines. It should also better explain the exclusion of financial firms and how financial-sector mechanisms affect BTD calculations.	income taxation in Vietnam are governed by distinct regulatory logics, with differences in deductible expenses, depreciation, provisions, tax incentives, and loss carry-forwards. We also discuss Circular No. 200/2014/TT-BTC and the role of tax incentives. In the Research Method section, we explain more explicitly why financial firms are excluded, including their distinct regulatory environments, balance sheet structures, income-generating mechanisms, loan-loss provisions, capital requirements, and tax treatments.	
2. There is no systematic sample selection and data collection procedure explaining how the final panel of 3,575 observations was derived from the initial population. It is unclear how data were extracted from audited reports or manually calculated.	We agree and have revised the Research Method section in detail. We now describe the initial population of 677 firms listed on HOSE and HNX as of 31 December 2023, the exclusion of financial firms, firms not applying Circular 200 after 2014, firms with extreme or inconsistent financial values, and firms without continuous data. We added Table 2, which reports the full sample selection process and shows how the final sample of 325 firms and 3,575 firm-year observations was obtained. We also clarified the manual data collection process, including direct extraction of pre-tax book income, operating cash flows, total assets, sales revenue, current tax expense, and manual calculation of BTDs and sales decline.	Research Method: Research design and data; Table 2 Sample selection counts.
3. The theoretical framework and hypotheses should be illustrated in a figure or flow chart. H1 and H2 feel disconnected and lack a theoretical lens, such as agency theory or signaling theory, explaining why sales decline should amplify the information content of BTDs.	Thank you for this helpful suggestion. We have revised the Theoretical background and literature review section to explicitly incorporate signaling theory, agency theory, and positive accounting theory. We have also added Fig 1, which presents the conceptual framework of the study. This figure illustrates how the theoretical lenses are linked to the key explanatory constructs, namely BTDs and sales decline, how earnings components operate as underlying transmission channels, and how these elements are connected to earnings	Theoretical background and literature review; Fig 1 Conceptual framework; Research hypotheses; Table 1 Summary of prior literature; Table 3 Empirical models, hypotheses, and expected coefficient signs.

	persistence and the four hypotheses. In the revised hypothesis development section, we further clarify how H1 and H2 form the baseline conditions for the integrated framework and how sales decline operates as a conditioning operating state. We also define the informational content and classification of LPBTD and LNBTD before deriving H3 and H4, so that the progression from H1 and H2 to the triple-interaction hypotheses is clearer and theoretically grounded. In addition, Table 3 is added as an empirical roadmap linking the hypotheses, focal coefficients, expected signs, and empirical result tables.	
4. The discussion provides weak conditional support through two-way interactions and does not critically address why the hypothesized three-way synergy did not materialize in Vietnam. The discussion should explain how Vietnamese institutional factors drive these results.	We agree and have substantially rewritten the Discussion. After re-estimating the regressions with firm-clustered standard errors and industry and year fixed effects, we interpret the results more cautiously. We now state that H2 is strongly supported, H1 receives partial and asymmetric support, and H3–H4 receive limited support because the three-way interaction terms are not statistically significant. We explain that the insignificant three-way effects may reflect the multiple sources of BTDs in Vietnam, including differences in accounting and tax rules, tax incentives, deductible expenses, depreciation, provisions, and loss carry-forwards. We also explain that sales declines may affect earnings persistence directly through cash-flow disruption, receivable collection, inventory adjustment, cost restructuring, and impairment recognition, not necessarily through BTD classifications alone.	Results and discussions: Discussion subsections “Book–tax differences and earnings persistence (H1),” “Sales decline and earnings persistence (H2),” and “Sales decline as a conditioning factor for BTD effects (H3 and H4).”
5. The conclusion is repetitive and lacks a clear statement on how the study advances accounting theory. Policy implications should provide actionable insights for auditors, tax authorities, and other stakeholders.	We revised the Conclusions and implications section. The revised version now states that the study contributes to accounting research by conceptualizing earnings persistence as a conditional attribute rather than a fixed property of reported earnings.	Conclusions, limitations, and future research: Conclusions and implications.

	We also explain practical implications for auditors, financial analysts, investors, tax authorities, and regulators. For example, BTDs during sales-decline periods may serve as risk indicators for assessing earnings sustainability, but should not be treated as direct evidence of non-compliance without firm-specific and industry-level information.	
6. The manuscript would benefit from final proofreading to ensure grammatical proficiency.	We revised the manuscript for grammar, clarity, and consistency. We also corrected spelling issues, including the abstract heading, standardized terminology, and improved consistency in the use of BTDs, PTBI, PTCF, PTACC, SD, EM, and TAXAD.	Entire manuscript.

3. Response to Reviewer 2

Reviewer 2 comment	Response	Revisions made and location in revised manuscript
1. Please further enhance the motivation to study this topic.	Thank you for this comment. We revised the Introduction to clarify the research motivation. The revised Introduction now explains that BTDs may arise from multiple sources and that interpreting them in isolation can be misleading, particularly during sales-decline periods. We also clarify why users of financial statements need to know when BTDs become more informative about the sustainability of current earnings.	Introduction.
2. Please clarify whether the statement that the adverse implications of BTDs arise predominantly through earnings management rather than tax avoidance applies to all Vietnamese listed firms or selected industries.	We revised the manuscript to avoid this overstatement. The revised analysis does not claim that the BTD–earnings persistence relation arises predominantly through earnings management rather than tax avoidance for all Vietnamese listed firms. Instead, the revised results are interpreted as association-based robustness evidence. We now state that the results remain informative after controlling for earnings management and tax avoidance, but we do not present these controls as causal channel tests.	Abstract; Results and discussions: Robustness; Conclusions and implications.
3. Please rectify citation errors showing "Error! Reference source not found."	We carefully checked and corrected all in-text citations and cross-references. The revised manuscript removes "Error! Reference source not found" messages and updates table, figure, and appendix references.	Entire manuscript; References.

4. Please add more recent studies from 2024 onward.	We added and discussed several recent studies, including Floropoulos et al. (2024), Rahiminejad (2024), Anderson and Rahiminejad (2025), and Le et al. (2025). These recent studies are used to strengthen the literature review, theoretical motivation, Vietnam-related evidence, and discussion.	Introduction; Theoretical background and literature review; Discussion; References.
5. Please provide a more comprehensive discussion of past studies and a summary of literature.	We added Table 1, which summarizes major research streams on BTDs, earnings persistence, institutional settings, sales decline, earnings components, and Vietnam-related evidence. We also expanded the literature discussion to clarify how the present study relates to prior research.	Theoretical background and literature review; Table 1 Summary of prior literature.
6. Please justify why panel data are used.	We added a paragraph explaining that panel data are appropriate because the research question requires linking current and one-year-ahead earnings for the same firm over time. The panel structure allows the analysis to exploit both cross-sectional variation in BTDs and time-series variation in sales conditions, while controlling for industry and year effects.	Research Method: Research design and data.
7. Please justify why the study uses annual data instead of quarterly reports.	We added a paragraph explaining that annual data are used because BTD construction requires current tax expense, pre-tax book income, and tax note information, which are more consistently available in audited annual reports. We also explain that quarterly reports in Vietnam generally provide less detailed tax-related information and may not allow consistent estimation of taxable income.	Research Method: Research design and data.
8. Please justify whether the study gives any treatment to the pandemic period because the sample includes 2014–2023.	We clarified that the study does not isolate COVID-19 as a separate event-study setting because the objective is not to estimate the specific pandemic effect. Instead, sales decline is treated as a firm-year operating state that may arise from multiple sources, including pandemic-related disruptions. Year fixed effects are included to control for common time-specific shocks. We also acknowledge this as a limitation and future research direction.	Research Method: Research design and data; Conclusions, limitations, and future research.
9. Please add diagnostic tests into the models.	We added diagnostic tests. Specifically, we conducted VIF tests for multicollinearity, Breusch–Pagan/Cook–Weisberg and White tests for heteroskedasticity, and Wooldridge tests for first-order serial correlation in panel data. The results are reported in Table 7. Based on these tests, all reported regressions are estimated with firm-clustered standard errors, and industry and year fixed effects are included.	Research Method: diagnostic tests paragraph; Results and discussions: Table 7 Diagnostic tests; Tables 8–12.

10. Please compare the findings with past studies.	We revised the Discussion to compare the findings with prior studies, including Lev and Nissim, Hanlon, Blaylock et al., Jackson, Atwood et al., Banker and Chen, Banker et al., Dechow and Ge, Anderson and Rahiminejad, and Floropoulos et al. The revised discussion now explains where the findings are consistent with prior research and where they refine or qualify prior evidence.	Results and discussions: Discussion subsections H1, H2, H3–H4.
11. Please add more justification on the study's implications.	We expanded the Conclusions and implications section. The revised section explains implications for auditors, financial analysts, investors, tax authorities, and regulators. It also clarifies that BTDs should be used as screening or risk indicators rather than direct evidence of non-compliance.	Conclusions, limitations, and future research: Conclusions and implications.
12. Please revise the format to comply with PLOS ONE, especially referencing format.	We revised the manuscript formatting and reference style. We checked the reference list, corrected citation errors, updated DOIs where available, removed duplicate or erroneous entries, and revised in-text citations into numerical format.	Entire manuscript; References.

4. **Response to Reviewer 3**

Reviewer 3 comment	Response	Revisions made and location in revised manuscript
1. The manuscript does not sufficiently articulate the incremental theoretical contribution of integrating BTD and sales decline. Please clarify the conditional perspective, why BTD provides incremental information after controlling for cash flows and accruals, and how Vietnam alters theoretical predictions.	Thank you for this important comment. We revised the Introduction and Theoretical background to clarify the incremental contribution. We now explain that the study adopts a conditional interpretation of BTDs, in which BTDs are treated as state-dependent signals rather than uniform proxies for earnings management or tax avoidance. We also clarify that cash flows and accruals capture the mechanical structure of earnings, while BTDs may contain additional information about accounting–tax differences, reporting choices, and institutional tax rules. Vietnam is now positioned as a context where accounting–tax separation, tax incentives, and operating volatility may affect the information content of BTDs.	Introduction; Theoretical background and literature review; Abstract.
2. Please further justify why re-examining BTD and earnings persistence is important in Vietnam. Provide institutional background on	We expanded the Vietnam institutional discussion in the Introduction. The revised manuscript explains differences between financial reporting and	Introduction; Results and discussions: Descriptive statistics; Table 5.

BTD, earnings management, or tax avoidance behavior, and explain how BTD affects earnings persistence during sales-decline years.	corporate income taxation in Vietnam, including deductible expenses, depreciation, provisions, tax incentives, and loss carry-forwards. We also added descriptive statistics showing variation in BTDs, LPBTD, LNBTD, EM, and TAXAD across sales-up and sales-down firm-years. These revisions strengthen the practical and institutional motivation for examining BTDs during sales-decline years.	
3. The hypotheses are incoherent and underdeveloped. H3 and H4 refer to triple interactions, but the progressive logic among H1–H4 is unclear. LPBTD and LNBTD are introduced abruptly. Please define BTD informational content and classification before deriving hypotheses.	We substantially revised the Research hypotheses section. Before developing H1–H4, we now define BTDs and clarify the classification of LPBTD and LNBTD. We also added a bridging paragraph explaining how H1 and H2 establish the baseline conditions for the integrated framework, and why H3 and H4 examine whether the persistence implications of BTDs differ during sales-decline periods. H3 and H4 have been rewritten to explain the distinct economic interpretations of LPBTD and LNBTD.	Theoretical background and literature review; Research hypotheses.
4. The interpretation of empirical results and mechanism tests is insufficiently deep. Most triple interaction terms are insignificant, but the paper provides no explanation or subgroup tests. The cash-flow and accrual decomposition is underdeveloped. Please discuss insignificant results and add heterogeneity tests by industry, firm size, and profitability.	We revised the empirical analysis and discussion in several ways. First, we re-estimated the models using OLS with firm-clustered standard errors and industry and year fixed effects. Second, we revised the discussion to explicitly state that the three-way interaction terms are not statistically significant, and therefore H3 and H4 receive limited support. Third, we explain why the hypothesized three-way effects may not appear uniformly in Vietnam, including multiple sources of BTDs and direct effects of sales decline on earnings persistence through cash-flow disruptions, inventory adjustments, receivable collection, cost restructuring, and impairments. Fourth, we clarified the component-level results by distinguishing the separate roles of sales decline, LPBTD, LNBTD, cash flows, and accruals. Regarding heterogeneity tests, we agree that this is an important extension. In the revised manuscript, we include industry and year fixed effects in all adjusted regressions and acknowledge in the limitations that the	Results and discussions: Tables 8–12; Discussion H3–H4; Conclusions, limitations, and future research: Limitations and Future research.

	study does not fully examine heterogeneity across firm size, profitability, or detailed industry groups. We identify this as an important direction for future research.	
5. Practical and policy implications are weak. Conclusions focus on statistical significance without deriving implications for investors, analysts, regulators, and tax authorities. The manuscript also overgeneralizes to other emerging or developed markets.	We revised the Abstract, Discussion, and Conclusions to avoid overgeneralization. The revised manuscript emphasizes that the evidence is context-specific to Vietnam and should not be generalized mechanically to other markets. We expanded the practical and policy implications for auditors, financial analysts, investors, tax authorities, and regulators. We explain that BTDs during sales-decline periods can serve as risk indicators for earnings sustainability, but should not be treated as direct evidence of tax non-compliance without firm-specific, industry-specific, and disclosure-based analysis.	Abstract; Conclusions, limitations, and future research: Conclusions and implications.

4. Additional Changes Made Beyond Reviewer-Specific Comments

Area revised	Revision made	Location in revised manuscript
Abstract	Revised to reflect the re-estimated results. The abstract now states that evidence for BTDs is asymmetric, that large positive BTDs are more consistently associated with lower earnings persistence, and that the three-way interaction terms are not statistically significant after firm-clustered standard errors.	Abstract.
Sample period and sample structure	Revised from the prior "unbalanced panel 2014–2023" description to a balanced panel of 325 non-financial listed firms from 2013 to 2023, corresponding to 3,575 firm-year observations.	Abstract; Research Method; Table 2.
Regression estimation	Re-estimated the main models using OLS with firm-clustered standard errors and industry and year fixed effects.	Research Method; Tables 8–12.
Diagnostic tests	Added VIF, Breusch–Pagan/Cook–Weisberg, White, and Wooldridge tests.	Research Method; Table 7.
Results interpretation	Revised the interpretation of H1–H4 to align with the re-estimated results: H2 is strongly supported; H1 receives partial and asymmetric support; H3 and H4 receive limited support.	Results and discussions.
Robustness interpretation	Revised the robustness discussion to avoid causal claims about earnings management and tax avoidance channels.	Results and discussions: Robustness.
Limitations and future research	Expanded limitations to include the use of public data, observational design, Vietnam-specific sample, COVID-19 treatment, and lack of detailed heterogeneity tests by size, profitability, or industry group.	Conclusions, limitations, and future research.

References	Added recent literature from 2024 onward and corrected reference formatting and DOI issues.	References.

Closing Statement

We are grateful to the Academic Editor and reviewers for their constructive comments, which helped us substantially improve the manuscript. We believe that the revised manuscript now provides a clearer motivation, a stronger theoretical framework, a more transparent research design, more rigorous diagnostic testing, a more cautious interpretation of the empirical results, and clearer theoretical, practical, and policy implications. We hope that the revised manuscript now meets the standards of PLOS ONE.

Sincerely,

Nguyen Ngoc Khanh Dung

(on behalf of all authors)
