# Supplementary material for: When do book–tax differences matter for earnings persistence? The role of sales decline
Source: PLoS One. 2026 Oct 28;21(10):e0344270. doi: 10.1371/journal.pone.0344270 (PMC13716203; doi:10.1371/journal.pone.0344270)
Supplement: Supplementary file 2 [file pone.0344270.s001.docx]

**S1 Appendix**

**Appendix A. Regression Specifications**

This appendix presents the complete set of regression equations that underpin the empirical framework summarized in Table 3. The specifications are reported to ensure transparency and facilitate replication.

Throughout, subscripts i and t denote firm and year, respectively. All income variables are scaled by total assets at the beginning of year t. Industry and year fixed effects are included in all adjusted specifications unless otherwise noted. Standard errors are clustered at the firm level in the reported regressions.

**1. Baseline earnings persistence**

The baseline model measures the persistence of pre-tax book income (PTBI):

PTBI_i,t+1_=β_0_+β_1_PTBI_i,t_+ε_i,t_	(A1)

The coefficient β1 captures earnings persistence.

**2. Sales decline as a conditioning variable**

To test whether earnings persistence differs during sales-decline periods, the following interaction model is estimated:

PTBI_i,t+1_=β_0_+β_1_PTBI_i,t_+β_2_SD_i,t_+β_3_(SD_i,t_×PTBI_i,t_)+ε_i,t_	(A2)

The interaction coefficient β3 measures the differential persistence of earnings during sales-decline periods. A negative estimate of β3 indicates lower earnings persistence when sales decline.

**3. Book–tax differences and earnings persistence**

To examine whether earnings persistence varies across BTD groups, PTBI is interacted with indicators for large negative and large positive BTDs:

PTBI_i,t+1_= β_0_+β_1_PTBI_i,t_+β_2_LNBTD_i,t_+β_3_LPBTD_i,t_

+ β_4_(LNBTD_i,t_×PTBI_i,t_)+β_5_(LPBTD_i,t_×PTBI_i,t_)+ε_i,t_	(A3)

The reference group consists of firm-year observations that are not classified as either LNBTD or LPBTD. The interaction coefficients β4 and β5 capture whether earnings persistence differs for firms with large negative and large positive BTDs, respectively, relative to the benchmark group.

**4. Integrated model: sales decline and BTD**

To test whether sales decline conditions the association between BTDs and earnings persistence, the following integrated specification is estimated:

PTBI_i,t+1_= 	β_0_+β_1_PTBI_i,t_+β_2_LNBTD_i,t_+β_3_LPBTD_i,t_+β_4_(LNBTD_i,t_×PTBI_i,t_)

+β_5_(LPBTD_i,t_×PTBI_i,t_)+β_6_SD_i,t_+β_7_(SD_i,t_×PTBI_i,t_)

+β_8_(LNBTD_i,t_× SD_i,t_) + β_9_ (LPBTD_i,t_ × SD_i,t_)

+ β_10_(LNBTD_i,t_×PTBI_i,t_×SD_i,t_)+β_11_(LPBTD_i,t_×PTBI_i,t_×SD_i,t_)+ε_i,t_	(A4)

The triple-interaction coefficients β10 and β11 capture whether the persistence of earnings associated with large negative and large positive BTDs, respectively, differs between sales-decline and non-sales-decline periods. Negative estimates of β10 and β11 are consistent with Hypotheses H4 and H3, respectively.

**5. Decomposition into cash flows and accruals**

Pre-tax book income is decomposed into pre-tax operating cash flows (PTCF) and pre-tax accruals (PTACC), where:

PTACC_i,t_=PTBI_i,t_−PTCF_i,t_

***5.1. Baseline component persistence***

PTBI_i,t+1_=β_0_+β_1_PTCF_i,t_+β_2_PTACC_i,t_+ε_i,t_	(A5)

The coefficients β1 and β2 capture the persistence of operating cash flows and accruals, respectively.

***5.2. Sales decline and earnings components***

PTBI_i,t+1_= 	β_0_+β_1_PTCF_i,t_+β_2_PTACC_i,t_+β_3_SD_i,t_

+ β_4_(SD_i,t_×PTCF_i,t_)+β_5_(SD_i,t_×PTACC_i,t_)+ε_i,t_	(A6)

The interaction coefficients β4 and β5 capture whether the persistence of operating cash flows and accruals differs during sales-decline periods.

***5.3. BTD and earnings components (without sales decline)***

PTBI_i,t+1_ = β_0_+β_1_PTCF_i,t_+β_2_PTACC_i,t_+β_3_LNBTD_i,t_

+ β_4_LPBTD_i,t_+β_5_(LNBTD_i,t_×PTCF_i,t_)+β_6_(LPBTD_i,t_×PTCF_i,t_)

+ β_7_(LNBTD_i,t_×PTACC_i,t_)+β_8_(LPBTD_i,t_×PTACC_i,t_)+ε_i,t_	(A7)

The interaction terms capture whether the persistence of operating cash flows and accruals differs across LNBTD and LPBTD firms relative to the benchmark group.

***5.4. Integrated component model: sales decline and BTD***

PTBI_i,t+1_= β_0_+β_1_PTCF_i,t_+β_2_PTACC_i,t_+β_3_LNBTD_i,t_

+β_4_LPBTD_i,t_+β_5_(LNBTD_i,t_×PTCF_i,t_)+β_6_(LPBTD_i,t_×PTCF_i,t_)

+ β_7_(LNBTD_i,t_×PTACC_i,t_)+β_8_(LPBTD_i,t_×PTACC_i,t_)

+ β_9_SD_i,t_+β_10_(SD_i,t_×PTCF_i,t_)+β_11_(SD_i,t_×PTACC_i,t_)

+ β_12_(SD_i,t_×LNBTD_i,t_)+β_13_(SD_i,t_×LPBTD_i,t_)

+ β_14_(SD_i,t_×LNBTD_i,t_×PTCF_i,t_)+β_15_(SD_i,t_×LPBTD_i,t_×PTCF_i,t_)

+ β_16_(SD_i,t_×LNBTD_i,t_×PTACC_i,t_)+β_17_(SD_i,t_×LPBTD_i,t_×PTACC_i,t_) +ε_i,t_	(A8)

The three-way interaction terms capture whether sales decline conditions the association between BTD groups and the persistence of operating cash flows and accruals.

**6. Extensions controlling for earnings management and tax avoidance**

The triple-interaction coefficients β10 and β11 capture whether the persistence of earnings associated with large negative and large positive BTDs, respectively, differs between sales-decline and non-sales-decline periods. Negative estimates of β10 and β11 are consistent with Hypotheses H4 and H3, respectively

PTBI_i,t+1_= β_0_+β_1_PTBI_i,t_+β_2_EM_i,t_+β_3_(EM_i,t_×PTBI_i,t_)+β_4_LNBTD_i,t_

+β_5_LPBTD_i,t_+β_6_(LNBTD_i,t_×PTBI_i,t_)+β_7_(LPBTD_i,t_×PTBI_i,t_)

+β_8_(EM_i,t_×LNBTD_i,t_)+β_9_(EM_i,t_×LPBTD_i,t_)

+ β_10_(EMi,t × LNBTDi,t × PTBIi,t) + β_11_(EM_i,t_ × LPBTD_i,t_ × PTBI_i,t_)

+β_12_SD_i,t_+β_13_(SD_i,t_×PTBI_i,t_)+β_14_(SD_i,t_×LNBTD_i,t_)

+β_15_(SD_i,t_×LPBTD_i,t_)+β_16_(SD_i,t_×LNBTD_i,t_×PTBI_i,t_)+

+ β_17_(SD_i,t_×LPBTD_i,t_×PTBI_i,t_) +ε_i,t_	(A9a)

PTBI_i,t+1_= β_0_+β_1_PTBI_i,t_+β_2_TAXAD_i,t_+β_3_(TAXAD_i,t_×PTBI_i,t_)+β_4_LNBTD_i,t_

+β_5_LPBTD_i,t_+β_6_(LNBTD_i,t_×PTBI_i,t_)+β_7_(LPBTD_i,t_×PTBI_i,t_)

+β_8_(TAXAD_i,t_×LNBTD_i,t_)+β_9_(TAXAD_i,t_×LPBTD_i,t_)

+β_10_SD_i,t_+β_11_(SD_i,t_×PTBI_i,t_)+β_12_(SD_i,t_×LNBTD_i,t_)

+β_13_(SD_i,t_×LPBTD_i,t_)+β_14_(SD_i,t_×LNBTD_i,t_×PTBI_i,t_)

+ β_15_(SD_i,t_×LPBTD_i,t_×PTBI_i,t_) +ε_i,t_	(A9b)
