# Supplementary material for: When do book–tax differences matter for earnings persistence? The role of sales decline
Source: PLoS One. 2026 Oct 28;21(10):e0344270. doi: 10.1371/journal.pone.0344270 (PMC13716203; doi:10.1371/journal.pone.0344270)
Supplement: Supplementary file 5 [file pone.0344270.s008.docx]

# Response to the Academic Editor and Reviewers

**Manuscript ID:** PONE-D-26-07956R2

**Title:** When do book–tax differences matter for earnings persistence? The role of sales decline

## Response to the Academic Editor

### 1. Summary

We thank the Academic Editor for recognizing the improvements made in the previous revision and for inviting us to address the remaining comments. We have revised the theoretical explanation, clarified the identification limits of the observational design, and reviewed citation and paragraph formatting. Detailed responses to Reviewer 3 are provided below.

### 2. Response to the Editor’s comment

**Comment E1:** “Following thorough revisions, the paper has been significantly improved. However, minor revisions are still needed for the paper to be accepted. Therefore, it is essential that the comments from Reviewer 3 are fully implemented.”

**Response:** Thank you for this guidance. We have clarified the distinct roles of signaling theory, agency theory, and positive accounting theory in Section 2.1 (pp. 8–10). We have also explained the specifications actually estimated and the possibility of reverse causality and time-varying confounding in Section 3.1 (p. 21), and added further robustness analyses using firm and year fixed effects, a common estimation sample, lagged firm-level controls, and lagged BTD indicators. These additions are described in Section 3.8 (pp. 26–27) and reported in the new Table 14 (pp. 50–52). We also revised the limitations and future-research sections (pp. 53–54). Finally, we have reviewed the citation format, references, and first-line indentation throughout the manuscript. Our responses to each of Reviewer 3’s comments follow.

## Response to Reviewer 2 Comments

### 1. Summary

We thank Reviewer 2 for reviewing the revised manuscript and for indicating that the previous comments have been addressed.

### 3. Point-by-point response to Comments and Suggestions for Authors

**Comment R2.1:** “All comments have been addressed.”

**Response:** Thank you for confirming that the comments from the previous review round have been addressed. We have retained those revisions. In the current revision, we have further clarified the distinction between the estimated associations and causal effects in response to Reviewer 3.

### 4. Response to Comments on the Quality of English Language

Reviewer 2 rated the manuscript’s presentation and English as satisfactory and identified no specific language issue.

### 5. Additional Clarifications

The clarification of identification limits does not change the reported regression estimates. It specifies more precisely what can be concluded from the observational analyses.

## Response to Reviewer 3 Comments

### 1. Summary

We thank Reviewer 3 for recognizing the relevance of our research question and for identifying three points requiring further attention. We have clarified the theoretical framework, made the possibility of reverse causality and time-varying confounding explicit, and reviewed the presentation of citations and paragraphs.

### 3. Point-by-point response to Comments and Suggestions for Authors

**Comment R3.1:** “The manuscript simultaneously invokes signaling theory, agency theory, and positive accounting theory to explain the BTD-earnings persistence relationship. However, the connections among these three theories and their respective roles across different hypotheses are not clearly specified. Signaling theory treats BTDs as externally observable signals, while agency theory links BTDs to managerial opportunism. These logics imply different underlying assumptions, but the paper does not adequately discuss the tensions or complementarities between them.”

**Response:** Thank you for identifying this ambiguity. We revised Section 2.1 to distinguish the interpretation of BTDs from the possible mechanisms generating them. The revised signaling-theory paragraph explains how external users may interpret an observable BTD (p. 8). The following paragraph assigns agency theory to the scope for managerial discretion and positive accounting theory to incentives that may shape accounting choices (p. 8). We also state explicitly that institutional or mechanical BTDs need not imply opportunistic behavior. A new paragraph maps H1 to differences in persistence across BTD groups, H2 to the direct association of sales decline with persistence, and H3–H4 to the additional interactions by BTD sign (p. 10). We interpret the unsupported three-way interactions consistently with this distinction in Section 4.7.3 (pp. 41–43).

**Revised text:** “These theories operate at different levels: signaling theory describes users’ interpretation; agency theory identifies the scope for discretion; and positive accounting theory identifies incentives for particular choices. They are complementary when discretion generates transitory differences, but institutional and mechanical BTDs need not involve managerial opportunism [5,13]. The observed association therefore cannot distinguish among these mechanisms by itself.” (Section 2.1, p. 8)

**Comment R3.2:** “Reverse causality may exist between BTDs and earnings persistence (e.g., firms expecting lower future earnings may engage in more tax planning or earnings management, affecting current BTDs), and omitted variables may confound the relationship. The current analysis primarily relies on panel fixed effects to control for unobserved heterogeneity, but fixed effects models only control for time-invariant omitted variables.”

**Response:** Thank you for raising this important identification concern. We agree that firms expecting weaker future performance may adjust their current reporting or tax-planning decisions, thereby affecting measured BTDs. Unobserved time-varying factors may also jointly influence current BTDs, sales decline, and future earnings.

We have revised the manuscript in two ways. First, we now clearly distinguish the main adjusted models from the additional firm fixed-effects models. The main adjusted specifications include industry and year indicators and firm-clustered standard errors. Industry indicators control for differences across industries, year indicators absorb shocks common to all firms, and clustering accounts for within-firm dependence in statistical inference. However, these adjustments do not eliminate reverse causality or unobserved time-varying confounding.

Second, we added the following analyses in the new Table 14:

1. firm and year fixed-effects models estimated using the full sample;
2. firm and year fixed-effects models estimated using the common sample available for the lagged-control specifications;
3. matched-sample models including one-year-lagged firm size, leverage, and asset tangibility; and
4. models replacing current BTD indicators and their corresponding interaction terms with one-year-lagged BTD indicators.

Standard errors are clustered by firm in all specifications.

In Panel A of Table 14, all three-way interaction coefficients are individually insignificant across the four specifications. The joint Wald tests are also insignificant, with p-values of 0.688, 0.910, 0.846, and 0.280, respectively. In Panel B, only the interaction between sales decline, lagged large negative BTDs, and accruals is individually significant. However, the four lagged three-way interactions are jointly insignificant (p = 0.180). We therefore do not interpret this isolated coefficient as consistent evidence supporting H3 or H4.

Overall, these analyses support the general conclusion that sales decline does not consistently amplify the association between BTD classifications and earnings persistence. Nevertheless, the additional analyses do not establish causality. Firm fixed effects absorb only time-invariant firm characteristics. Lagged controls account for selected observed characteristics, and lagged BTD indicators improve temporal ordering, but neither approach provides exogenous variation in BTDs or eliminates unobserved time-varying confounding. Accordingly, all interaction estimates are interpreted as conditional associations rather than causal effects.

**Revised text in Section 3.1:** “The main adjusted specifications include industry and year indicators and firm-clustered standard errors. Industry indicators control for time-invariant differences across industries, while year indicators absorb shocks common to all firms. Because these adjustments do not eliminate unobserved firm heterogeneity or reverse causality, we conduct additional sensitivity analyses using firm and year fixed effects, a common estimation sample, lagged firm-level controls, and lagged BTD indicators. These tests mitigate concerns regarding time-invariant firm characteristics, sample comparability, and temporal ordering, but they do not identify an exogenous change in BTDs or eliminate unobserved time-varying confounding. Accordingly, all estimated interaction effects are interpreted as conditional associations rather than causal effects.”

**Additional text in Section 3.8:** “Finally, to address concerns regarding firm heterogeneity, sample comparability, and temporal ordering, we estimate additional specifications reported in Table 14. These analyses employ firm and year fixed effects with standard errors clustered by firm. We first estimate the models using the full sample and then re-estimate them on the common sample available for the lagged-control specifications. The third specification includes one-year-lagged firm size, leverage, and asset tangibility. The final specification replaces the current BTD indicators and their associated interaction terms with one-year-lagged BTD indicators. These analyses are treated as sensitivity tests rather than causal identification strategies.”

**Revised interpretation of Table 14:** “Table 14 presents additional analyses addressing firm heterogeneity, sample comparability, and temporal ordering. Across the firm fixed-effects, matched-sample, lagged-control, and lagged-BTD specifications, the general conclusion regarding H3 and H4 remains unchanged. In Panel A, none of the three-way interactions is individually significant, and the corresponding joint Wald tests are insignificant in all four specifications. In Panel B, only the interaction between sales decline, lagged LNBTD, and accruals is significant. However, the four lagged three-way interactions are jointly insignificant (p = 0.180). This isolated coefficient therefore does not provide consistent support for H3 or H4. Although these analyses mitigate concerns regarding omitted time-invariant firm characteristics and strengthen temporal ordering, they do not eliminate reverse causality or unobserved time-varying confounding.”

**Revised limitation:** “Finally, although the additional analyses employ firm and year fixed effects, a common estimation sample, lagged controls, and lagged BTD indicators, they do not fully eliminate reverse causality or unobserved time-varying confounding. Firm fixed effects absorb time-invariant firm characteristics, whereas lagged variables improve temporal ordering; neither approach provides exogenous variation in BTDs. Firms may still adjust their reporting or tax positions in anticipation of future operating performance. The results should therefore be interpreted as conditional associations rather than causal effects.”

**Revised future-research direction:** “Future research could use tax-return data, administrative records, or more detailed firm-level tax disclosures to validate taxable-income estimates and identify the underlying sources of BTDs. Stronger causal identification could be obtained from credible instruments, regulatory changes, tax-policy discontinuities, or other quasi-experimental settings. Future studies could also incorporate richer time-varying controls, alternative measures of earnings management and tax avoidance, longer panels, and cross-country samples.”

**Location in the revised manuscript:**

- Section 3.1, p. 21: revised discussion of reverse causality, omitted variables, and causal limitations.
- Section 3.8, pp. 26–27: description of the firm fixed-effects, matched-sample, lagged-control, and lagged-BTD analyses.
- Table 14 and accompanying discussion, pp. 50–52: presentation and interpretation of the additional results.
- Section 5.2, pp. 53–54: expanded discussion of reverse causality and time-varying confounding.
- Section 5.3, p. 54: revised future-research directions.

**Comment R3.3:** “It is recommended that the manuscript adopt a uniform reference citation format. ([2,3,19,20] [14][17]. and [5],[13]) Please thoroughly check the manuscript for trivial formatting errors such as misplaced double brackets like [13]]. And standardize the first-line indentation format throughout the manuscript.”

**Response:** Thank you for drawing our attention to these inconsistencies. We reviewed the complete manuscript and standardized the numbered in-text citation format. Multiple references are now reported within a single pair of square brackets, for example, [1,2] instead of [1],[2]; [8,9] instead of [8][9]; and [24,25,31–35] instead of [24],[25],[31]-[35].

We also corrected misplaced or duplicated brackets and standardized first-line indentation across the body paragraphs. In addition, we standardized decimal notation and thousands separators, table notes and significance symbols, incomplete variable definitions, cross-references, Appendix equations, and reference-list formatting. Inaccurate bibliographic information and the duplicate reference entry were corrected, and the corresponding citation numbers were updated. These presentation corrections do not affect the empirical estimates or conclusions.

**Location in the revised manuscript:**

- Pages 3–54: in-text citations, paragraph indentation, headings, table notes, and numerical notation standardized throughout.
- Table 3, pp. 28–30: variable definitions and cross-references corrected.
- Table 5, pp. 31–33: decimal notation and thousands separators standardized.
- Table 14, pp. 50–52: table labels, numerical notation, p-value presentation, and notes standardized.
- References, pp. 55–58: bibliographic entries corrected and standardized.
- Appendix A, pp. 59–61: equation formatting and duplicated mathematical symbols corrected.

### 4. Response to Comments on the Quality of English Language

Reviewer 3 rated the manuscript’s English as satisfactory and identified no separate language issue. We thank the reviewer and have reviewed the wording of the revised passages.

### 5. Additional Clarifications

The revisions clarify the theoretical interpretation and the limits of inference. The new analyses in Table 14 supplement the previously reported models and do not change the general conclusion regarding the lack of consistent support for H3 and H4. We have checked that the descriptions of the main and robustness results correspond to the estimates reported in Tables 8–14.

# Concluding Response

We again sincerely thank the Academic Editor and the Reviewers for their careful and constructive comments. The revisions have improved the theoretical clarity, empirical transparency, and presentation of the manuscript. We hope that the revised manuscript is now suitable for publication in *PLOS ONE*.

Sincerely,

**The Authors**
