# Supplementary material for: When do book–tax differences matter for earnings persistence? The role of sales decline
Source: PLoS One. 2026 Oct 28;21(10):e0344270. doi: 10.1371/journal.pone.0344270 (PMC13716203; doi:10.1371/journal.pone.0344270)
Supplement: Supplementary file 6 [file pone.0344270.s004.docx]

Review report

This study examines earnings persistence in relation to book–tax differences (BTD) and sales decline among non-financial listed firms in Vietnam. Although it is an interesting and meaningful paper, there are some further revisions as follows.

1.Issues of incremental contribution. Although prior studies have extensively examined the relationship between BTD and earnings persistence (e.g., Hanlon, 2005; Blaylock et al., 2012) as well as the moderating role of sales decline (e.g., Anderson & Rahiminejad, 2025), this manuscript does not sufficiently articulate the unique theoretical contribution of integrating these elements into a unified framework. The authors should more clearly clarify in the introduction and theory section: (i) the essential theoretical distinction between the conditional perspective adopted here and those used in previous research; (ii) why BTD continues to provide incremental information after controlling for cash flows and accruals, and what this finding implies for or challenges in the existing literature; and (iii) how the institutional characteristics of Vietnam—such as its high accounting-tax separation and pronounced operating volatility—specifically alter theoretical predictions regarding the BTD-earnings persistence relationship.

2. Issue of Adequacy and Justification of Sample.Using Vietnamese listed firms as the research setting is acceptable, but the authors need to further justify why re-examining the relationship between BTD and earnings persistence is particularly important in emerging markets such as Vietnam. It is recommended that the authors provide institutional background on the distribution of BTD, earnings management, or tax avoidance behaviors in Vietnam (e.g., tax incentives, enforcement intensity), and explain how BTD affects earnings persistence during years of sales decline, so as to strengthen the practical motivation of the study.

3.Issues of hypotheses development.The theoretical chain of H1–H4 is incoherent and underdeveloped with insufficient explanation of moderating mechanisms. H3 and H4 refer to triple interaction effects, but the progressive logical relationship among all hypotheses is not clearly clarified. In particular, the introduction of LPBTD and LNBTD in H3–H4 is abrupt and lacks prior motivation. The authors should clearly define the informational content and classification of BTDs before deriving hypotheses. Furthermore, the reasoning for all four hypotheses—especially H3–H4, which are overly simplistic—should be strengthened to improve clarity, rigor, and consistency.

4. Issues of research design. The interpretation of empirical results and mechanism tests is insufficiently deep. Most triple interaction terms are statistically insignificant, yet the paper provides no reasonable explanation or subgroup tests. Moreover, the analysis of cash flow and accrual decomposition is underdeveloped, failing to explain why LNBTD functions mainly through the cash flow channel while LPBTD acts through both cash flow and accrual channels. It is suggested that the authors discuss the insignificant results and add heterogeneity tests by industry, firm size, and profitability.

5. Issues of Practical and Policy Implications.The practical and policy implications of this study are relatively weak. The conclusions only focus on statistical significance without deriving clear implications for investors, financial analysts, regulators, and tax authorities. Although the findings are based on Vietnamese data, the authors repeatedly imply generalizability to other emerging markets and even developed markets in the abstract and conclusion; however, such discussions remain overly vague and underdeveloped.

References:

Hanlon M. The persistence and pricing of earnings, accruals, and cash flows when firms have large book–tax differences. The Accounting Review. 2005;80(1):137–166. https://doi.org/10.2308/accr.2005.80.1.137

Blaylock B, Shevlin T, Wilson RJ. Tax avoidance, large positive temporary book–tax differences, and earnings persistence. The Accounting Review. 2012;87(1):91– 120. https://doi.org/10.2308/accr-10158

Anderson M, Rahiminejad S. Book–tax differences and earnings persistence: The conditioning role of sales decline. Journal of Corporate Accounting & Finance. 2025;36(1):1– 22. https://doi.org/10.1002/jcaf.22760
