# Supplementary material for: When do book–tax differences matter for earnings persistence? The role of sales decline
Source: PLoS One. 2026 Oct 28;21(10):e0344270. doi: 10.1371/journal.pone.0344270 (PMC13716203; doi:10.1371/journal.pone.0344270)
Supplement: Supplementary file 7 [file pone.0344270.s007.docx]

Response to Reviewers

Manuscript ID: PONE-D-26-07956R1

Title: When Do Book–Tax Differences Matter for Earnings Persistence? The Role of Sales Decline

Dear Academic Editor and Reviewers,

We sincerely thank the Academic Editor and the three reviewers for their constructive and detailed comments on our manuscript. We have carefully revised the manuscript to address all comments. The revised version substantially strengthens the theoretical framework, institutional motivation, hypothesis development, sample selection, variable measurement, research design, robustness analyses, interpretation of empirical findings, practical implications, limitations, and English presentation. We also corrected and updated the reference list, reviewed the terminology used throughout the manuscript, improved the consistency between the theoretical framework and the empirical evidence, and performed additional robustness analyses using alternative measures of sales decline, an alternative BTD classification, and a subsample excluding the COVID-19 period.

All changes have been incorporated into the revised manuscript and highlighted in the tracked-changes version. Below, we provide a point-by-point response.

# 1. Response to Academic Editor

## Comment 1. The DOI provided for References 25 and 5 is incorrect. In addition, the DOI provided for Reference 6 (Noga & Schnader, 2013) cannot be found. Please carefully review and update the reference list to ensure that all references contain accurate bibliographic information and correct DOIs.

**Response:** We have thoroughly reviewed the entire reference list and corrected all identified errors. Specifically, we corrected the DOI for References 5 and 25, updated the bibliographic information for Reference 6 (including the correct page range and DOI), and rechecked all remaining references to ensure that the bibliographic information and DOI links are accurate and consistent throughout the manuscript.

**Revisions made and location in revised manuscript:** The reference list has been updated.

# 2. Response to Reviewer 3

## 1. Insufficient theoretical engagement with the null results. H3 and H4 predict that sales decline amplifies the negative BTD-persistence association. The empirical results do not support this. The manuscript attributes the null findings to "institutional complexity" and "multiple sources of BTDs." However, they do not consider whether the underlying theory itself may need refinement.

**Response:** Thank you for this insightful comment. We agree that our original discussion focused primarily on institutional complexity and the heterogeneous sources of BTDs without sufficiently reconsidering the theoretical mechanism underlying H3 and H4. We have therefore revised both the theoretical framework (Section 2.1) and the Discussion (Section 4.7.3). Specifically, we now distinguish the direct operating effect of sales decline from a BTD-specific amplification effect. The revised manuscript argues that sales decline directly weakens earnings persistence through operating disruptions, whereas an incremental amplification effect would be expected only when the underlying economic sources of BTDs are systematically related to the accounting or operational adjustments induced by declining sales. Accordingly, the insignificant three-way interactions are interpreted as evidence that the proposed mechanism is contingent on the economic source of BTDs rather than an automatic consequence of adverse operating conditions. We also revised the Conclusions to ensure consistency with this refined theoretical interpretation.

**Revisions made and location in revised manuscript:** Sections 2.1, 4.7.3, and 5.1.

## 2. Lack of theoretical explanation for LPBTD vs. LNBTD asymmetry. The finding that LPBTD is robustly associated with lower persistence while LNBTD shows unstable evidence is both interesting and theoretically important. Yet the manuscript does not provide a systematic theoretical account for this asymmetry in the Vietnamese context. One possibility is that Vietnam's tax incentive regime (e.g., "four-years exemption, nine-years half-rate") generates predominantly positive BTDs, making LPBTD more reflective of institutional features than opportunistic behavior. The manuscript should develop and test such context-specific explanations.

**Response:** Thank you for this insightful comment. We agree that the original manuscript did not provide a sufficiently developed theoretical explanation for the asymmetric findings between LPBTDs and LNBTDs. In response, we have substantially strengthened the theoretical framework by incorporating a context-specific institutional explanation based on Vietnam's preferential corporate income tax regimes. The revised manuscript explains that tax incentives based on industry, location, and investment projects may legitimately generate positive book–tax differences even in the absence of opportunistic reporting. Consequently, LPBTDs may reflect institutional characteristics embedded in Vietnam's tax system as well as accounting–tax divergence, making them a more homogeneous and stable signal than LNBTDs. By contrast, LNBTDs are argued to arise from a broader range of firm-specific economic events, including conservative accounting recognition, impairment losses, restructuring costs, non-deductible expenses, and other temporary adjustments, resulting in greater heterogeneity and less stable associations with earnings persistence.

We also incorporated this institutional explanation into the Discussion to interpret the stronger empirical evidence for LPBTDs than for LNBTDs. At the same time, we explicitly acknowledge that this interpretation cannot be tested directly because publicly available financial statements do not consistently disclose firm-level tax incentive status throughout the sample period. This limitation has been added to the manuscript, and future research is encouraged to combine financial statement data with detailed tax incentive information to examine this explanation directly.

**Revisions made and location in revised manuscript:**

- **Section 2.2 (Research hypotheses):** Expanded the theoretical development immediately following the introductory discussion on the classification of LPBTDs and LNBTDs (before Section 2.2.1).
- **Section 4.7.3 (Sales decline as a conditioning factor for BTD effects):** Added a context-specific discussion explaining the asymmetric empirical findings between LPBTDs and LNBTDs.
- **Sections 5.2 and 5.3:** Added the corresponding limitation regarding the unavailability of firm-level tax incentive information and suggested future research using detailed tax incentive data to directly examine the proposed institutional explanation.

## 3. Insufficient justification for proxy variable validity. Taxable income is estimated by dividing current tax expense by statutory tax rate instead of direct disclosure in financial statements; the paper does not verify the reliability of this estimation method under Vietnamese accounting data. Earnings management and tax avoidance only adopt single proxies without alternative robustness tests. Sales decline is merely a binary dummy, lacking a continuous measure of decline magnitude for comparative analysis.

**Response:** Thank you for this valuable comment. We acknowledge that taxable income is not publicly disclosed in Vietnamese financial statements, making direct validation against tax-return data infeasible. We therefore revised the manuscript to clarify the rationale for estimating taxable income from current corporate income tax expense divided by the applicable statutory corporate income tax rate and explicitly acknowledge that the resulting BTD measure should be interpreted as an approximation rather than an exact measure of taxable income. We also clarify that this approach follows the standard methodology adopted in the BTD literature when taxable income is unavailable.

We further revised the Method section to clarify that earnings management and tax avoidance are included primarily as additional control variables to mitigate omitted-variable bias rather than as the primary constructs of interest. Their use as single proxies is now explicitly acknowledged as a limitation, and future research employing alternative proxy measures is recommended.

In response to the comment regarding sales decline, we additionally conducted a robustness analysis using a continuous measure of sales decline (SDMAG), defined as the absolute magnitude of negative annual sales growth. This alternative specification produced qualitatively similar results to the baseline analyses, providing additional support for the stability of the main conclusions.

**Revisions made and location in revised manuscript:** Sections 3.3, 3.4, 3.7, 3.8, 5.2, and Table 13.

## 4. Weak robustness design. The paper only controls EM and tax avoidance as additional covariates, without alternative dependent variables (next-period operating cash flow), alternative BTD quantile grouping or subsample regression excluding the COVID period for cross-validation, threatening the stability of core conclusions.

**Response:**

Thank you for this constructive suggestion. We have substantially expanded the robustness analyses in the revised manuscript. First, we re-estimated the models using a continuous measure of sales decline (SDMAG) instead of the baseline binary indicator. Second, we re-estimated all fully specified models after excluding observations from the COVID-19 period (2020–2021). Third, we employed a more stringent classification of large book–tax differences by defining LPBTD10 and LNBTD10 as the highest and lowest annual deciles of the BTD distribution rather than the baseline annual quintiles. Across these additional analyses, the principal conclusions remained qualitatively unchanged. Specifically, sales decline continued to be associated with lower earnings persistence, LPBTDs remained more consistently associated with lower earnings persistence than LNBTDs, and the hypothesized three-way interaction between sales decline, BTDs, and current earnings remained generally unsupported. These additional robustness analyses strengthen the reliability of the baseline findings.

**Revisions made and location in revised manuscript:** Section 3.8, Section 4.8 (Robustness tests), and Table 13.

# Closing Statement

We are grateful to the Academic Editor and reviewers for their constructive comments, which have substantially improved the quality of this manuscript. In response to the comments, we refined the theoretical framework, strengthened the institutional motivation, revised the hypothesis development, clarified the measurement of key variables, expanded the robustness analyses, improved the consistency between the theoretical arguments and the empirical evidence, corrected and updated the reference list, and carefully reviewed the English language and terminology throughout the manuscript. We believe that these revisions have substantially strengthened the manuscript and hope that the revised version is now suitable for publication in PLOS ONE.

Sincerely,

Nguyen Ngoc Khanh Dung

(on behalf of all authors)
