# Supplementary material for: When do book–tax differences matter for earnings persistence? The role of sales decline
Source: PLoS One. 2026 Oct 28;21(10):e0344270. doi: 10.1371/journal.pone.0344270 (PMC13716203; doi:10.1371/journal.pone.0344270)
Supplement: Peer review history

# Peer review: Decision Letter 0

**DOI:** 10.1371/journal.pone.0344270.r001

**Authors:** Thomas Kollruss

19 Apr 2026

PONE-D-26-07956

When Do Book–Tax Differences Matter for Earnings Persistence? The Role of Sales Decline

PLOS One?

Dear Dr. Dung,

Thank you for submitting your manuscript to PLOS ONE. After careful consideration, we feel that it has merit but does not fully meet PLOS ONE’s publication criteria as it currently stands. Therefore, we invite you to submit a revised version of the manuscript that addresses the points raised during the review process.

Please submit your revised manuscript by May 28 2026 11:59PM. If you will need more time than this to complete your revisions, please reply to this message or contact the journal office at plosone@plos.org. When you're ready to submit your revision, log on to https://www.editorialmanager.com/pone/ and select the 'Submissions Needing Revision' folder to locate your manuscript file.

- A letter that responds to each point raised by the academic editor and reviewer(s). You should upload this letter as a separate file labeled 'Response to Reviewers'.
- A marked-up copy of your manuscript that highlights changes made to the original version. You should upload this as a separate file labeled 'Revised Manuscript with Track Changes'.
- An unmarked version of your revised paper without tracked changes. You should upload this as a separate file labeled 'Manuscript'.

If you would like to make changes to your financial disclosure, please include your updated statement in your cover letter. Guidelines for resubmitting your figure files are available below the reviewer comments at the end of this letter.

If applicable, we recommend that you deposit your laboratory protocols in protocols.io to enhance the reproducibility of your results. Protocols.io assigns your protocol its own identifier (DOI) so that it can be cited independently in the future. For instructions see: https://journals.plos.org/plosone/s/submission-guidelines#loc-laboratory-protocols. Additionally, PLOS ONE offers an option for publishing peer-reviewed Lab Protocol articles, which describe protocols hosted on protocols.io. Read more information on sharing protocols at https://plos.org/protocols?utm_medium=editorial-email&utm_source=authorletters&utm_campaign=protocols.

As the corresponding author, your ORCID iD is verified in the submission system and will appear in the published article. PLOS supports the use of ORCID, and we encourage all coauthors to register for an ORCID iD and use it as well. Please encourage your coauthors to verify their ORCID iD within the submission system before final acceptance, as unverified ORCID iDs will not appear in the published article. *Only*  the individual author can complete the verification step; PLOS staff *cannot*  verify ORCID iDs on behalf of authors.

We look forward to receiving your revised manuscript.

Kind regards,

Prof. Dr. Thomas Kollruss

Academic Editor

PLOS One

**Journal Requirements:**

1. When submitting your revision, we need you to address these additional requirements.

Please ensure that your manuscript meets PLOS ONE's style requirements, including those for file naming. The PLOS ONE style templates can be found at

https://journals.plos.org/plosone/s/file?id=wjVg/PLOSOne_formatting_sample_main_body.pdf and

https://journals.plos.org/plosone/s/file?id=ba62/PLOSOne_formatting_sample_title_authors_affiliations.pdf

2. For studies involving third-party data, we encourage authors to share any data specific to their analyses that they can legally distribute. PLOS recognizes, however, that authors may be using third-party data they do not have the rights to share. When third-party data cannot be publicly shared, authors must provide all information necessary for interested researchers to apply to gain access to the data. (https://journals.plos.org/plosone/s/data-availability#loc-acceptable-data-access-restrictions)

For any third-party data that the authors cannot legally distribute, they should include the following information in their Data Availability Statement upon submission:

a) A description of the data set and the third-party source

b) If applicable, verification of permission to use the data set

c) Confirmation of whether the authors received any special privileges in accessing the data that other researchers would not have

d) All necessary contact information others would need to apply to gain access to the data

3. Your ethics statement should only appear in the Methods section of your manuscript. If your ethics statement is written in any section besides the Methods, please move it to the Methods section and delete it from any other section. Please ensure that your ethics statement is included in your manuscript, as the ethics statement entered into the online submission form will not be published alongside your manuscript.

4. We have noted some discrepancies in the references and bibliography. Particularly, reference (30) appears to be missing, reference (12) has been repeated and 'error! Reference not found!' appears several times in the manuscript text. Further, the DOI provided for reference (23) Banker RD, Byzalov D, Fang S, Liang Y. Cost management research. Journal of Management Accounting Research. 2017;29(3):1–25, does not appear to match the title of the article. Additionally, the DOI provided for reference (6) T. J. Noga, A. L. Schnader. Book-tax differences as an indicator of financial distress. Accounting Horizons. cannot be found.

Could you please update your manuscript carefully to ensure all references are listed properly with their appropriate DOIs and the error message has been removed? We thank you for your attention to these requests.

5. If the reviewer comments include a recommendation to cite specific previously published works, please review and evaluate these publications to determine whether they are relevant and should be cited. There is no requirement to cite these works unless the editor has indicated otherwise.

**Additional Editor Comments:**

This paper requires thorough revision. All of the reviewers’ comments must be fully addressed. In particular, the introduction needs to be more detailed and precise. The study’s limitations must be described (in greater detail). The practical implications of the findings, and the need for further research, must be outlined in greater detail.

[Note: HTML markup is below. Please do not edit.]

Reviewers' comments:

Reviewer's Responses to Questions

**Comments to the Author**

1. Is the manuscript technically sound, and do the data support the conclusions?

Reviewer #1: Partly

Reviewer #2: Partly

Reviewer #3: Yes

**********

2. Has the statistical analysis been performed appropriately and rigorously?

Reviewer #1: No

Reviewer #2: No

Reviewer #3: Yes

**********

3. Have the authors made all data underlying the findings in their manuscript fully available?

The PLOS Data policy

Reviewer #1: No

Reviewer #2: Yes

Reviewer #3: Yes

**********

4. Is the manuscript presented in an intelligible fashion and written in standard English?

Reviewer #1: No

Reviewer #2: Yes

Reviewer #3: Yes

**********

**Reviewer #1:**  Thank you for the efforts. I am pleased to have a chance to be a reviewer of this manuscript. This study investigates the conditional nature of earnings persistence by examining the interplay between Book-Tax Differences (BTDs) and sales decline within the context of the Vietnamese emerging market. While the topic is relevant and the use of a 10-year panel (2014–2023) provides a robust dataset, the manuscript currently suffers from significant structural and methodological gaps. I have some major comments as follows:

1. Introduction: Please consider addressing the reason why the context of Vietnam is significant to represent for the topic of accounting-tax separation and significant revenue volatility. I recommend the author dedicate a specific subsection in the Introduction or Institutional Background to explicitly argue why the Vietnamese regulatory framework makes BTD a particularly informative signal during sales declines. Also, I might agree with the authors for exclusion the financial firms due to their unique regulatory environments and balance sheet structures, which render earnings persistence measures incomparable to non-financial entities. However, the manuscript would benefit from a more explicit discussion on how these unique mechanisms specifically affect BTD calculations in the Vietnamese context.

2. There is no systematic sample selection and data collection to describe the details how the final unbalanced panel of 3,575 observations was derived from the initial population. It is unclear how specific data points were directly extracted from audited reports or if any manual adjustments/calculation were made.

3. It should be better to illustrate the theoretical framework and hypothesis in the figure/flow chart. The development of H1 (BTD) and H2 (Sales Decline) feels disconnected. It is lacking theoretical lens (e.g., Agency Theory or Signaling Theory) that convincingly explains why an operating shock like sales decline should mechanically amplify the information content of BTDs.

4. Results and discussion: The author made huge efforts to present the conditional support through two-way interactions, which is a weak interpretation. The discussion fails to critically address why the hypothesized three-way synergy did not materialize in the Vietnamese context. As an emerging market study, there is insufficient discussion on how Vietnamese-specific institutional factors (e.g., specific tax laws or accounting-tax conformity levels) drive these specific results.

5. The conclusion is a repetitive summary of the findings. It lacks a clear statement on how this study advances accounting theory. The policy implication should provide actionable insights for stakeholder, such as how auditors or tax authorities should flag BTDs during revenue contractions.

6. Language: Even I am a native English speaker, and the English is generally clear. However, the manuscript would benefit from a final proofread to ensure the grammatical proficiency.

**Reviewer #2:**  General notes

The paper studies on When Do Book–Tax Differences Matter for Earnings Persistence? The Role of Sales Decline. This paper can be further furnish by taking into considering of following issues.

Main issues

1. Suggestion: Please further enhance motivation to study this topic.

2. Suggestion: Please further clarify following statement

during periods of sales decline, the adverse implications of BTDs for earnings persistence arise predominantly through the earnings management channel rather than tax avoidance.

is represent whole Vietnamese listed firms or for selected industries?

3. Suggestion: Please rectify some citation show Error! Reference source not found.

4. Suggestion: Please add on more citation Year 2024 onward past studies in this paper.

5. Suggestion: Please provide add more comprehensive discussion of past studies and summary of literature.

6. Suggestion: Please justify panel data method is used.

7. Suggestion: Please justify this study uses annual data instead of quarterly report.

8. Suggestion: Please justify this study gives any treatment in pandemic period since this study include from 2014 to 2023.

9. Suggestion: Please add in diagnostic tests into models.

10. Suggestion: Please add past studies findings compare with findings of this study.

11. Suggestion: Please add on more justification on this study’s implication.

Overall comments

Generally, this is an interesting research topic.

Please revise format of paper to comply with format of PLOS One. Example referencing format.

**Reviewer #3:**  Please refer to the attached file.

This study examines earnings persistence in relation to book–tax differences (BTD) and sales decline among non-financial listed firms in Vietnam. Although it is an interesting and meaningful paper, there are some further revisions as follows.

1.Issues of incremental contribution. Although prior studies have extensively examined the relationship between BTD and earnings persistence (e.g., Hanlon, 2005; Blaylock et al., 2012) as well as the moderating role of sales decline (e.g., Anderson & Rahiminejad, 2025), this manuscript does not sufficiently articulate the unique theoretical contribution of integrating these elements into a unified framework. The authors should more clearly clarify in the introduction and theory section: (i) the essential theoretical distinction between the conditional perspective adopted here and those used in previous research; (ii) why BTD continues to provide incremental information after controlling for cash flows and accruals, and what this finding implies for or challenges in the existing literature; and (iii) how the institutional characteristics of Vietnam—such as its high accounting-tax separation and pronounced operating volatility—specifically alter theoretical predictions regarding the BTD-earnings persistence relationship.

2. Issue of Adequacy and Justification of Sample.Using Vietnamese listed firms as the research setting is acceptable, but the authors need to further justify why re-examining the relationship between BTD and earnings persistence is particularly important in emerging markets such as Vietnam. It is recommended that the authors provide institutional background on the distribution of BTD, earnings management, or tax avoidance behaviors in Vietnam (e.g., tax incentives, enforcement intensity), and explain how BTD affects earnings persistence during years of sales decline, so as to strengthen the practical motivation of the study.

3.Issues of hypotheses development.The theoretical chain of H1–H4 is incoherent and underdeveloped with insufficient explanation of moderating mechanisms. H3 and H4 refer to triple interaction effects, but the progressive logical relationship among all hypotheses is not clearly clarified. In particular, the introduction of LPBTD and LNBTD in H3–H4 is abrupt and lacks prior motivation. The authors should clearly define the informational content and classification of BTDs before deriving hypotheses. Furthermore, the reasoning for all four hypotheses—especially H3–H4, which are overly simplistic—should be strengthened to improve clarity, rigor, and consistency.

4. Issues of research design. The interpretation of empirical results and mechanism tests is insufficiently deep. Most triple interaction terms are statistically insignificant, yet the paper provides no reasonable explanation or subgroup tests. Moreover, the analysis of cash flow and accrual decomposition is underdeveloped, failing to explain why LNBTD functions mainly through the cash flow channel while LPBTD acts through both cash flow and accrual channels. It is suggested that the authors discuss the insignificant results and add heterogeneity tests by industry, firm size, and profitability.

5. Issues of Practical and Policy Implications.The practical and policy implications of this study are relatively weak. The conclusions only focus on statistical significance without deriving clear implications for investors, financial analysts, regulators, and tax authorities. Although the findings are based on Vietnamese data, the authors repeatedly imply generalizability to other emerging markets and even developed markets in the abstract and conclusion; however, such discussions remain overly vague and underdeveloped.

References:

Hanlon M. The persistence and pricing of earnings, accruals, and cash flows when firms have large book–tax differences. The Accounting Review. 2005;80(1):137–166. https://doi.org/10.2308/accr.2005.80.1.137

Blaylock B, Shevlin T, Wilson RJ. Tax avoidance, large positive temporary book–tax differences, and earnings persistence. The Accounting Review. 2012;87(1):91– 120. https://doi.org/10.2308/accr-10158

Anderson M, Rahiminejad S. Book–tax differences and earnings persistence: The conditioning role of sales decline. Journal of Corporate Accounting & Finance. 2025;36(1):1– 22. https://doi.org/10.1002/jcaf.22760

**********

what does this mean?). If published, this will include your full peer review and any attached files.

If you choose “no”, your identity will remain anonymous but your review may still be made public.

**Do you want your identity to be public for this peer review?**  For information about this choice, including consent withdrawal, please see our Privacy Policy

Reviewer #1: No

Reviewer #2: No

Reviewer #3: No

**********

[NOTE: If reviewer comments were submitted as an attachment file, they will be attached to this email and accessible via the submission site. Please log into your account, locate the manuscript record, and check for the action link "View Attachments". If this link does not appear, there are no attachment files.]

To ensure your figures meet our technical requirements, please review our figure guidelines: https://journals.plos.org/plosone/s/figures

You may also use PLOS’s free figure tool, NAAS, to help you prepare publication quality figures: https://journals.plos.org/plosone/s/figures#loc-tools-for-figure-preparation.

NAAS will assess whether your figures meet our technical requirements by comparing each figure against our figure specifications.

Attachment Submitted filename: Review report.docx

# Author comment: Author response to Decision Letter 1

**DOI:** 10.1371/journal.pone.0344270.r002

19 Jun 2026

1. Response to Academic Editor

Comment 1. The paper requires thorough revision. All reviewers’ comments must be fully addressed. The introduction needs to be more detailed and precise. The study’s limitations must be described in greater detail. The practical implications and future research need to be outlined in greater detail.

Response: We thank the Academic Editor for this guidance. We have thoroughly revised the manuscript. The Introduction now provides a clearer motivation, explains why Vietnam is an appropriate institutional setting, clarifies the conditional perspective adopted in the study, and avoids broad generalization beyond the Vietnamese setting. The Conclusions, limitations, and future research section has also been substantially expanded to clarify theoretical contributions, practical implications, limitations, and future research directions.

Revisions made and location in revised manuscript: Introduction; Theoretical background and literature review; Research Method; Results and discussions; Conclusions, limitations, and future research.

Comment 2. Please ensure that the manuscript meets PLOS ONE style requirements, including referencing format.

Response: We revised the manuscript to comply more closely with PLOS ONE formatting requirements. The reference list has been checked and reformatted using numerical citation style. We corrected prior citation errors and removed “Error! Reference source not found” messages.

Revisions made and location in revised manuscript: References; in-text citations throughout the manuscript.

Comment 3. For studies involving third-party data, please provide a Data Availability Statement with sufficient information.

Response: We clarified that the data are derived from publicly available annual financial statements and corporate reports of listed firms from HOSE, HNX, and firms’ official disclosures. We also prepared the dataset and Stata code for reproducibility.

Revisions made and location in revised manuscript: Data availability statement. Before final submission, we will ensure that the dataset and Stata codes are either provided as Supporting Information or that all access information to public data sources is fully specified in accordance with PLOS ONE’s data policy.

Comment 4. Ethics statement should appear only in the Methods section.

Response: We revised the ethics statement to clarify that the study uses secondary publicly available corporate financial statement data and does not involve human participants, personal data, confidential information, animal subjects, or field research.

Revisions made and location in revised manuscript: Ethics statement in the manuscript.

Comment 5. References and bibliography contain discrepancies, including missing reference, repeated reference, DOI issues, and “Error! Reference source not found.”

Response: We carefully checked the reference list and in-text citations. We removed citation errors, corrected the repeated reference issue, added missing references, and updated the DOI information where available. We also added recent studies from 2024 onward, including Floropoulos et al. (2024), Rahiminejad (2024), Anderson and Rahiminejad (2025), and Le et al. (2025).

Revisions made and location in revised manuscript: References; Introduction; Theoretical background; Discussion.

2. Response to Reviewer 1

1. The Introduction should explain why Vietnam is significant for accounting–tax separation and revenue volatility. The manuscript should explicitly argue why the Vietnamese regulatory framework makes BTD a particularly informative signal during sales declines. It should also better explain the exclusion of financial firms and how financial-sector mechanisms affect BTD calculations.

Response: Thank you for this important comment. We have substantially revised the Introduction to explain why Vietnam is an appropriate institutional setting. We now explain that financial reporting and corporate income taxation in Vietnam are governed by distinct regulatory logics, with differences in deductible expenses, depreciation, provisions, tax incentives, and loss carry-forwards. We also discuss Circular No. 200/2014/TT-BTC and the role of tax incentives. In the Research Method section, we explain more explicitly why financial firms are excluded, including their distinct regulatory environments, balance sheet structures, income-generating mechanisms, loan-loss provisions, capital requirements, and tax treatments.

Revisions made and location in revised manuscript: Introduction: paragraph on Vietnam institutional setting. Research Method: Research design and data; Table 2 Sample selection counts.

2. There is no systematic sample selection and data collection procedure explaining how the final panel of 3,575 observations was derived from the initial population. It is unclear how data were extracted from audited reports or manually calculated.

Response: We agree and have revised the Research Method section in detail. We now describe the initial population of 677 firms listed on HOSE and HNX as of 31 December 2023, the exclusion of financial firms, firms not applying Circular 200 after 2014, firms with extreme or inconsistent financial values, and firms without continuous data. We added Table 2, which reports the full sample selection process and shows how the final sample of 325 firms and 3,575 firm-year observations was obtained. We also clarified the manual data collection process, including direct extraction of pre-tax book income, operating cash flows, total assets, sales revenue, current tax expense, and manual calculation of BTDs and sales decline.

Revisions made and location in revised manuscript: Research Method: Research design and data; Table 2 Sample selection counts.

3. The theoretical framework and hypotheses should be illustrated in a figure or flow chart. H1 and H2 feel disconnected and lack a theoretical lens, such as agency theory or signaling theory, explaining why sales decline should amplify the information content of BTDs.

Response: Thank you for this helpful suggestion. We have revised the Theoretical background and literature review section to explicitly incorporate signaling theory, agency theory, and positive accounting theory. We have also added Fig 1, which presents the conceptual framework of the study. This figure illustrates how the theoretical lenses are linked to the key explanatory constructs, namely BTDs and sales decline, how earnings components operate as underlying transmission channels, and how these elements are connected to earnings persistence and the four hypotheses. In the revised hypothesis development section, we further clarify how H1 and H2 form the baseline conditions for the integrated framework and how sales decline operates as a conditioning operating state. We also define the informational content and classification of LPBTD and LNBTD before deriving H3 and H4, so that the progression from H1 and H2 to the triple-interaction hypotheses is clearer and theoretically grounded. In addition, Table 3 is added as an empirical roadmap linking the hypotheses, focal coefficients, expected signs, and empirical result tables.

Revisions made and location in revised manuscript: Theoretical background and literature review; Fig 1 Conceptual framework; Research hypotheses; Table 1 Summary of prior literature; Table 3 Empirical models, hypotheses, and expected coefficient signs.

4. The discussion provides weak conditional support through two-way interactions and does not critically address why the hypothesized three-way synergy did not materialize in Vietnam. The discussion should explain how Vietnamese institutional factors drive these results.

Response: We agree and have substantially rewritten the Discussion. After re-estimating the regressions with firm-clustered standard errors and industry and year fixed effects, we interpret the results more cautiously. We now state that H2 is strongly supported, H1 receives partial and asymmetric support, and H3–H4 receive limited support because the three-way interaction terms are not statistically significant. We explain that the insignificant three-way effects may reflect the multiple sources of BTDs in Vietnam, including differences in accounting and tax rules, tax incentives, deductible expenses, depreciation, provisions, and loss carry-forwards. We also explain that sales declines may affect earnings persistence directly through cash-flow disruption, receivable collection, inventory adjustment, cost restructuring, and impairment recognition, not necessarily through BTD classifications alone.

Revisions made and location in revised manuscript: Results and discussions: Discussion subsections “Book–tax differences and earnings persistence (H1),” “Sales decline and earnings persistence (H2),” and “Sales decline as a conditioning factor for BTD effects (H3 and H4).”

5. The conclusion is repetitive and lacks a clear statement on how the study advances accounting theory. Policy implications should provide actionable insights for auditors, tax authorities, and other stakeholders.

Response: We revised the Conclusions and implications section. The revised version now states that the study contributes to accounting research by conceptualizing earnings persistence as a conditional attribute rather than a fixed property of reported earnings. We also explain practical implications for auditors, financial analysts, investors, tax authorities, and regulators. For example, BTDs during sales-decline periods may serve as risk indicators for assessing earnings sustainability, but should not be treated as direct evidence of non-compliance without firm-specific and industry-level information.

Revisions made and location in revised manuscript: Conclusions, limitations, and future research: Conclusions and implications.

6. The manuscript would benefit from final proofreading to ensure grammatical proficiency.

Response: We revised the manuscript for grammar, clarity, and consistency. We also corrected spelling issues, including the abstract heading, standardized terminology, and improved consistency in the use of BTDs, PTBI, PTCF, PTACC, SD, EM, and TAXAD.

Revisions made and location in revised manuscript: Entire manuscript.

3. Response to Reviewer 2

1. Please further enhance the motivation to study this topic.

Response: Thank you for this comment. We revised the Introduction to clarify the research motivation. The revised Introduction now explains that BTDs may arise from multiple sources and that interpreting them in isolation can be misleading, particularly during sales-decline periods. We also clarify why users of financial statements need to know when BTDs become more informative about the sustainability of current earnings.

Revisions made and location in revised manuscript: Introduction.

2. Please clarify whether the statement that the adverse implications of BTDs arise predominantly through earnings management rather than tax avoidance applies to all Vietnamese listed firms or selected industries.

Response: We revised the manuscript to avoid this overstatement. The revised analysis does not claim that the BTD–earnings persistence relation arises predominantly through earnings management rather than tax avoidance for all Vietnamese listed firms. Instead, the revised results are interpreted as association-based robustness evidence. We now state that the results remain informative after controlling for earnings management and tax avoidance, but we do not present these controls as causal channel tests.

Revisions made and location in revised manuscript: Abstract; Results and discussions: Robustness; Conclusions and implications.

3. Please rectify citation errors showing “Error! Reference source not found.”

Response: We carefully checked and corrected all in-text citations and cross-references. The revised manuscript removes “Error! Reference source not found” messages and updates table, figure, and appendix references.

Revisions made and location in revised manuscript: Entire manuscript; References.

4. Please add more recent studies from 2024 onward.

Response: We added and discussed several recent studies, including Floropoulos et al. (2024), Rahiminejad (2024), Anderson and Rahiminejad (2025), and Le et al. (2025). These recent studies are used to strengthen the literature review, theoretical motivation, Vietnam-related evidence, and discussion.

Revisions made and location in revised manuscript: Introduction; Theoretical background and literature review; Discussion; References.

5. Please provide a more comprehensive discussion of past studies and a summary of literature.

Response: We added Table 1, which summarizes major research streams on BTDs, earnings persistence, institutional settings, sales decline, earnings components, and Vietnam-related evidence. We also expanded the literature discussion to clarify how the present study relates to prior research.

Revisions made and location in revised manuscript: Theoretical background and literature review; Table 1 Summary of prior literature.

6. Please justify why panel data are used.

Response: We added a paragraph explaining that panel data are appropriate because the research question requires linking current and one-year-ahead earnings for the same firm over time. The panel structure allows the analysis to exploit both cross-sectional variation in BTDs and time-series variation in sales conditions, while controlling for industry and year effects.

Revisions made and location in revised manuscript: Research Method: Research design and data.

7. Please justify why the study uses annual data instead of quarterly reports.

Response: We added a paragraph explaining that annual data are used because BTD construction requires current tax expense, pre-tax book income, and tax note information, which are more consistently available in audited annual reports. We also explain that quarterly reports in Vietnam generally provide less detailed tax-related information and may not allow consistent estimation of taxable income.

Revisions made and location in revised manuscript: Research Method: Research design and data.

8. Please justify whether the study gives any treatment to the pandemic period because the sample includes 2014–2023.

Response: We clarified that the study does not isolate COVID-19 as a separate event-study setting because the objective is not to estimate the specific pandemic effect. Instead, sales decline is treated as a firm-year operating state that may arise from multiple sources, including pandemic-related disruptions. Year fixed effects are included to control for common time-specific shocks. We also acknowledge this as a limitation and future research direction.

Revisions made and location in revised manuscript: Research Method: Research design and data; Conclusions, limitations, and future research.

9. Please add diagnostic tests into the models.

Response: We added diagnostic tests. Specifically, we conducted VIF tests for multicollinearity, Breusch–Pagan/Cook–Weisberg and White tests for heteroskedasticity, and Wooldridge tests for first-order serial correlation in panel data. The results are reported in Table 7. Based on these tests, all reported regressions are estimated with firm-clustered standard errors, and industry and year fixed effects are included.

Revisions made and location in revised manuscript: Research Method: diagnostic tests paragraph; Results and discussions: Table 7 Diagnostic tests; Tables 8–12.

10. Please compare the findings with past studies.

Response: We revised the Discussion to compare the findings with prior studies, including Lev and Nissim, Hanlon, Blaylock et al., Jackson, Atwood et al., Banker and Chen, Banker et al., Dechow and Ge, Anderson and Rahiminejad, and Floropoulos et al. The revised discussion now explains where the findings are consistent with prior research and where they refine or qualify prior evidence.

Revisions made and location in revised manuscript: Results and discussions: Discussion subsections H1, H2, H3–H4.

11. Please add more justification on the study’s implications.

Response: We expanded the Conclusions and implications section. The revised section explains implications for auditors, financial analysts, investors, tax authorities, and regulators. It also clarifies that BTDs should be used as screening or risk indicators rather than direct evidence of non-compliance.

Revisions made and location in revised manuscript: Conclusions, limitations, and future research: Conclusions and implications.

12. Please revise the format to comply with PLOS ONE, especially referencing format.

Response: We revised the manuscript formatting and reference style. We checked the reference list, corrected citation errors, updated DOIs where available, removed duplicate or erroneous entries, and revised in-text citations into numerical format.

Revisions made and location in revised manuscript: Entire manuscript; References.

4. Response to Reviewer 3

1. The manuscript does not sufficiently articulate the incremental theoretical contribution of integrating BTD and sales decline. Please clarify the conditional perspective, why BTD provides incremental information after controlling for cash flows and accruals, and how Vietnam alters theoretical predictions.

Response: Thank you for this important comment. We revised the Introduction and Theoretical background to clarify the incremental contribution. We now explain that the study adopts a conditional interpretation of BTDs, in which BTDs are treated as state-dependent signals rather than uniform proxies for earnings management or tax avoidance. We also clarify that cash flows and accruals capture the mechanical structure of earnings, while BTDs may contain additional information about accounting–tax differences, reporting choices, and institutional tax rules. Vietnam is now positioned as a context where accounting–tax separation, tax incentives, and operating volatility may affect the information content of BTDs.

Revisions made and location in revised manuscript: Introduction; Theoretical background and literature review; Abstract.

2. Please further justify why re-examining BTD and earnings persistence is important in Vietnam. Provide institutional background on BTD, earnings management, or tax avoidance behavior, and explain how BTD affects earnings persistence during sales-decline years.

Response: We expanded the Vietnam institutional discussion in the Introduction. The revised manuscript explains differences between financial reporting and corporate income taxation in Vietnam, including deductible expenses, depreciation, provisions, tax incentives, and loss carry-forwards. We also added descriptive statistics showing variation in BTDs, LPBTD, LNBTD, EM, and TAXAD across sales-up and sales-down firm-years. These revisions strengthen the practical and institutional motivation for examining BTDs during sales-decline years.

Revisions made and location in revised manuscript: Introduction; Results and discussions: Descriptive statistics; Table 5.

3. The hypotheses are incoherent and underdeveloped. H3 and H4 refer to triple interactions, but the progressive logic among H1–H4 is unclear. LPBTD and LNBTD are introduced abruptly. Please define BTD informational content and classification before deriving hypotheses.

Response: We substantially revised the Research hypotheses section. Before developing H1–H4, we now define BTDs and clarify the classification of LPBTD and LNBTD. We also added a bridging paragraph explaining how H1 and H2 establish the baseline conditions for the integrated framework, and why H3 and H4 examine whether the persistence implications of BTDs differ during sales-decline periods. H3 and H4 have been rewritten to explain the distinct economic interpretations of LPBTD and LNBTD.

Revisions made and location in revised manuscript: Theoretical background and literature review; Research hypotheses.

4. The interpretation of empirical results and mechanism tests is insufficiently deep. Most triple interaction terms are insignificant, but the paper provides no explanation or subgroup tests. The cash-flow and accrual decomposition is underdeveloped. Please discuss insignificant results and add heterogeneity tests by industry, firm size, and profitability.

Response: We revised the empirical analysis and discussion in several ways. First, we re-estimated the models using OLS with firm-clustered standard errors and industry and year fixed effects. Second, we revised the discussion to explicitly state that the three-way interaction terms are not statistically significant, and therefore H3 and H4 receive limited support. Third, we explain why the hypothesized three-way effects may not appear uniformly in Vietnam, including multiple sources of BTDs and direct effects of sales decline on earnings persistence through cash-flow disruptions, inventory adjustments, receivable collection, cost restructuring, and impairments. Fourth, we clarified the component-level results by distinguishing the separate roles of sales decline, LPBTD, LNBTD, cash flows, and accruals. Regarding heterogeneity tests, we agree that this is an important extension. In the revised manuscript, we include industry and year fixed effects in all adjusted regressions and acknowledge in the limitations that the study does not fully examine heterogeneity across firm size, profitability, or detailed industry groups. We identify this as an important direction for future research.

Revisions made and location in revised manuscript: Results and discussions: Tables 8–12; Discussion H3–H4; Conclusions, limitations, and future research: Limitations and Future research.

5. Practical and policy implications are weak. Conclusions focus on statistical significance without deriving implications for investors, analysts, regulators, and tax authorities. The manuscript also overgeneralizes to other emerging or developed markets.

Response: We revised the Abstract, Discussion, and Conclusions to avoid overgeneralization. The revised manuscript emphasizes that the evidence is context-specific to Vietnam and should not be generalized mechanically to other markets. We expanded the practical and policy implications for auditors, financial analysts, investors, tax authorities, and regulators. We explain that BTDs during sales-decline periods can serve as risk indicators for earnings sustainability, but should not be treated as direct evidence of tax non-compliance without firm-specific, industry-specific, and disclosure-based analysis.

Revisions made and location in revised manuscript: Abstract; Conclusions, limitations, and future research: Conclusions and implications.

5. Additional Changes Made Beyond Reviewer-Specific Comments

Additional change 1. Abstract

Revision made: Revised to reflect the re-estimated results. The abstract now states that evidence for BTDs is asymmetric, that large positive BTDs are more consistently associated with lower earnings persistence, and that the three-way interaction terms are not statistically significant after firm-clustered standard errors.

Location in revised manuscript: Abstract.

Additional change 2. Sample period and sample structure

Revision made: Revised from the prior “unbalanced panel 2014–2023” description to a balanced panel of 325 non-financial listed firms from 2013 to 2023, corresponding to 3,575 firm-year observations.

Location in revised manuscript: Abstract; Research Method; Table 2.

Additional change 3. Regression estimation

Revision made: Re-estimated the main models using OLS with firm-clustered standard errors and industry and year fixed effects.

Location in revised manuscript: Research Method; Tables 8–12.

Additional change 4. Diagnostic tests

Revision made: Added VIF, Breusch–Pagan/Cook–Weisberg, White, and Wooldridge tests.

Location in revised manuscript: Research Method; Table 7.

Additional change 5. Results interpretation

Revision made: Revised the interpretation of H1–H4 to align with the re-estimated results: H2 is strongly supported; H1 receives partial and asymmetric support; H3 and H4 receive limited support.

Location in revised manuscript: Results and discussions.

Additional change 6. Robustness interpretation

Revision made: Revised the robustness discussion to avoid causal claims about earnings management and tax avoidance channels.

Location in revised manuscript: Results and discussions: Robustness.

Additional change 7. Limitations and future research

Revision made: Expanded limitations to include the use of public data, observational design, Vietnam-specific sample, COVID-19 treatment, and lack of detailed heterogeneity tests by size, profitability, or industry group.

Location in revised manuscript: Conclusions, limitations, and future research.

Additional change 8. References

Revision made: Added recent literature from 2024 onward and corrected reference formatting and DOI issues.

Location in revised manuscript: References.

Attachment Submitted filename: Letter_Plos1.pdf

# Peer review: Decision Letter 1

**DOI:** 10.1371/journal.pone.0344270.r003

**Authors:** Thomas Kollruss

14 Jul 2026

PONE-D-26-07956R1

When do book–tax differences matter for earnings persistence? The role of sales decline

PLOS One?

Dear Dr. Dung,

As mentioned in point 4 of the previous revision letter, we have noted some discrepancies in the references and bibliography. Despite revision there are concerns regarding the folloiwng references: the DOI provided for reference (25) Banker RD, Byzalov D, Fang S, Liang Y. Cost management research. Journal of Management Accounting Research. 2017;29(3):1–25, is incorrect. Additionally, the DOI provided for reference (6) T. J. Noga, A. L. Schnader. Book-tax differences as an indicator of financial distress. Accounting Horizons. cannot be found For reference 5, Atwood TJ, Drake MS, Myers LA. Book–tax conformity, earnings persistence and the association between earnings and cash flows. Journal of Accounting and Economics. 2010;50(1):111–125, the DOI provided is incorrect.

Could you please update your manuscript carefully to ensure all references are listed properly with their appropriate DOIs and the error message has been removed? We thank you for your attention to these requests

Please submit your revised manuscript by August 5, 2026. If you will need more time than this to complete your revisions, please reply to this message or contact the journal office at plosone@plos.org. When you're ready to submit your revision, log on to https://www.editorialmanager.com/pone/ and select the 'Submissions Needing Revision' folder to locate your manuscript file.

Please include the following items when submitting your revised manuscript:

- A letter that responds to each point raised by the academic editor and reviewer(s). You should upload this letter as a separate file labeled 'Response to Reviewers'.
- A marked-up copy of your manuscript that highlights changes made to the original version. You should upload this as a separate file labeled 'Revised Manuscript with Track Changes'.
- An unmarked version of your revised paper without tracked changes. You should upload this as a separate file labeled 'Manuscript'

If you would like to make changes to your financial disclosure, please include your updated statement in your cover letter. Guidelines for resubmitting your figure files are available below the reviewer comments at the end of this letter.

If applicable, we recommend that you deposit your laboratory protocols in protocols.io to enhance the reproducibility of your results. Protocols.io assigns your protocol its own identifier (DOI) so that it can be cited independently in the future. For instructions see: https://journals.plos.org/plosone/s/submission-guidelines#loc-laboratory-protocols. Additionally, PLOS ONE offers an option for publishing peer-reviewed Lab Protocol articles, which describe protocols hosted on protocols.io. Read more information on sharing protocols at https://plos.org/protocols?utm_medium=editorial-email&utm_source=authorletters&utm_campaign=protocols.

As the corresponding author, your ORCID iD is verified in the submission system and will appear in the published article. PLOS supports the use of ORCID, and we encourage all coauthors to register for an ORCID iD and use it as well. Please encourage your coauthors to verify their ORCID iD within the submission system before final acceptance, as unverified ORCID iDs will not appear in the published article. *Only*  the individual author can complete the verification step; PLOS staff *cannot*  verify ORCID iDs on behalf of authors.

We look forward to receiving your revised manuscript.

Kind regards,

Prof. Dr. Thomas Kollruss

Academic Editor

PLOS One

Journal Requirements:

If the reviewer comments include a recommendation to cite specific previously published works, please review and evaluate these publications to determine whether they are relevant and should be cited. There is no requirement to cite these works unless the editor has indicated otherwise.

Please review your reference list to ensure that it is complete and correct. If you have cited papers that have been retracted, please include the rationale for doing so in the manuscript text, or remove these references and replace them with relevant current references. Any changes to the reference list should be mentioned in the rebuttal letter that accompanies your revised manuscript. If you need to cite a retracted article, indicate the article’s retracted status in the References list and also include a citation and full reference for the retraction notice.

Reviewers' comments:

Reviewer's Responses to Questions

**Comments to the Author**

Reviewer #2: All comments have been addressed

Reviewer #3: (No Response)

**********

2. Is the manuscript technically sound, and do the data support the conclusions?

Reviewer #2: Partly

Reviewer #3: Partly

**********

3. Has the statistical analysis been performed appropriately and rigorously?

Reviewer #2: Yes

Reviewer #3: Yes

**********

4. Have the authors made all data underlying the findings in their manuscript fully available?

The PLOS Data policy

Reviewer #2: Yes

Reviewer #3: Yes

**********

5. Is the manuscript presented in an intelligible fashion and written in standard English?

Reviewer #2: Yes

Reviewer #3: Yes

**********

Reviewer #2: The paper studies When do book–tax differences matter for earnings persistence? The role of sales decline.

Feedback

1. Revise is done.

2. Suggestion: Please proof read before publish.

Reviewer #3: Review report

This manuscript adopts a balanced panel dataset of Vietnamese non-financial listed firms from 2013 to 2023 and investigates the joint impact of book-tax differences (BTDs) and sales decline on earnings persistence from a conditional perspective. However, the manuscript contains some flaws in theoretical logic, empirical identification, statistical testing, result interpretation and writing standards.

1.Insufficient theoretical engagement with the null results. H3 and H4 predict that sales decline amplifies the negative BTD-persistence association. The empirical results do not support this. The manuscript attributes the null findings to "institutional complexity" and "multiple sources of BTDs." However, they do not consider whether the underlying theory itself may need refinement.

2.Lack of theoretical explanation for LPBTD vs. LNBTD asymmetry. The finding that LPBTD is robustly associated with lower persistence while LNBTD shows unstable evidence is both interesting and theoretically important. Yet the manuscript does not provide a systematic theoretical account for this asymmetry in the Vietnamese context. One possibility is that Vietnam's tax incentive regime (e.g., "four-years exemption, nine-years half-rate") generates predominantly positive BTDs, making LPBTD more reflective of institutional features than opportunistic behavior. The manuscript should develop and test such context-specific explanations.

3.Insufficient justification for proxy variable validity. Taxable income is estimated by dividing current tax expense by statutory tax rate instead of direct disclosure in financial statements; the paper does not verify the reliability of this estimation method under Vietnamese accounting data. Earnings management and tax avoidance only adopt single proxies without alternative robustness tests. Sales decline is merely a binary dummy, lacking a continuous measure of decline magnitude for comparative analysis.

4.Weak robustness design. The paper only controls EM and tax avoidance as additional covariates, without alternative dependent variables (next-period operating cash flow), alternative BTD quantile grouping or subsample regression excluding the COVID period for cross-validation, threatening the stability of core conclusions.

**********

what does this mean?). If published, this will include your full peer review and any attached files.

If you choose “no”, your identity will remain anonymous but your review may still be made public.

**Do you want your identity to be public for this peer review?**  For information about this choice, including consent withdrawal, please see our Privacy Policy

Reviewer #2: No

Reviewer #3: No

**********

[NOTE: If reviewer comments were submitted as an attachment file, they will be attached to this email and accessible via the submission site. Please log into your account, locate the manuscript record, and check for the action link "View Attachments". If this link does not appear, there are no attachment files.]

To ensure your figures meet our technical requirements, please review our figure guidelines: https://journals.plos.org/plosone/s/figures

You may also use PLOS’s free figure tool, NAAS, to help you prepare publication quality figures: https://journals.plos.org/plosone/s/figures#loc-tools-for-figure-preparation.

NAAS will assess whether your figures meet our technical requirements by comparing each figure against our figure specifications.

# Author comment: Author response to Decision Letter 2

**DOI:** 10.1371/journal.pone.0344270.r004

4 Aug 2026

We sincerely thank the Academic Editor and the three reviewers for their constructive and detailed comments on our manuscript. We have carefully revised the manuscript to address all comments. The revised version substantially strengthens the theoretical framework, institutional motivation, hypothesis development, sample selection, variable measurement, research design, robustness analyses, interpretation of empirical findings, practical implications, limitations, and English presentation. We also corrected and updated the reference list, reviewed the terminology used throughout the manuscript, improved the consistency between the theoretical framework and the empirical evidence, and performed additional robustness analyses using alternative measures of sales decline, an alternative BTD classification, and a subsample excluding the COVID-19 period.

All changes have been incorporated into the revised manuscript and highlighted in the tracked-changes version. Below, we provide a point-by-point response.

Attachment Submitted filename: 03.08_Letter_Plos_text_response_PONE-D-26-07956R1.docx

# Peer review: Decision Letter 2

**DOI:** 10.1371/journal.pone.0344270.r005

**Authors:** Thomas Kollruss

17 Aug 2026

PONE-D-26-07956R2

When do book–tax differences matter for earnings persistence? The role of sales decline

PLOS One

Dear Dr. Nguyen

**Following thorough revisions, the paper has been significantly improved. However, minor revisions are still needed for the paper to be accepted. Therefore, it is essential that the comments from Reviewer 3 are fully implemented (see Reviewer #3: Review report below).**

Please submit your revised manuscript by Oct 01 2026 11:59PM. If you will need more time than this to complete your revisions, please reply to this message or contact the journal office at plosone@plos.org. When you're ready to submit your revision, log on to https://www.editorialmanager.com/pone/ and select the 'Submissions Needing Revision' folder to locate your manuscript file.

- A letter that responds to each point raised by the academic editor and reviewer(s). You should upload this letter as a separate file labeled 'Response to Reviewers'.
- A marked-up copy of your manuscript that highlights changes made to the original version. You should upload this as a separate file labeled 'Revised Manuscript with Track Changes'.
- An unmarked version of your revised paper without tracked changes. You should upload this as a separate file labeled 'Manuscript'.

If you would like to make changes to your financial disclosure, please include your updated statement in your cover letter. Guidelines for resubmitting your figure files are available below the reviewer comments at the end of this letter.

If applicable, we recommend that you deposit your laboratory protocols in protocols.io to enhance the reproducibility of your results. Protocols.io assigns your protocol its own identifier (DOI) so that it can be cited independently in the future. For instructions see: https://journals.plos.org/plosone/s/submission-guidelines#loc-laboratory-protocols. Additionally, PLOS ONE offers an option for publishing peer-reviewed Lab Protocol articles, which describe protocols hosted on protocols.io. Read more information on sharing protocols at https://plos.org/protocols?utm_medium=editorial-email&utm_source=authorletters&utm_campaign=protocols.

As the corresponding author, your ORCID iD is verified in the submission system and will appear in the published article. PLOS supports the use of ORCID, and we encourage all coauthors to register for an ORCID iD and use it as well. Please encourage your coauthors to verify their ORCID iD within the submission system before final acceptance, as unverified ORCID iDs will not appear in the published article. *Only*  the individual author can complete the verification step; PLOS staff *cannot*  verify ORCID iDs on behalf of authors.

We look forward to receiving your revised manuscript.

Kind regards,

Prof. Dr. Thomas Kollruss

Academic Editor

PLOS One

Journal Requirements:

If the reviewer comments include a recommendation to cite specific previously published works, please review and evaluate these publications to determine whether they are relevant and should be cited. There is no requirement to cite these works unless the editor has indicated otherwise.

Please review your reference list to ensure that it is complete and correct. If you have cited papers that have been retracted, please include the rationale for doing so in the manuscript text, or remove these references and replace them with relevant current references. Any changes to the reference list should be mentioned in the rebuttal letter that accompanies your revised manuscript. If you need to cite a retracted article, indicate the article’s retracted status in the References list and also include a citation and full reference for the retraction notice.

Reviewers' comments:

Reviewer's Responses to Questions

**Comments to the Author**

Reviewer #2: All comments have been addressed

Reviewer #3: (No Response)

**********

2. Is the manuscript technically sound, and do the data support the conclusions?

Reviewer #2: Partly

Reviewer #3: Yes

**********

3. Has the statistical analysis been performed appropriately and rigorously?

Reviewer #2: Yes

Reviewer #3: Yes

**********

4. Have the authors made all data underlying the findings in their manuscript fully available?

The PLOS Data policy

Reviewer #2: Yes

Reviewer #3: Yes

**********

5. Is the manuscript presented in an intelligible fashion and written in standard English?

Reviewer #2: Yes

Reviewer #3: Yes

**********

Reviewer #2: (No Response)

Reviewer #3: Review report

This paper examines the association between book-tax differences (BTDs), sales decline, and earnings persistence among non-financial listed firms in Vietnam, a timely and theoretically relevant topic. However, the paper still has some weaknesses as follows.

1.The manuscript simultaneously invokes signaling theory, agency theory, and positive accounting theory to explain the BTD-earnings persistence relationship. However, the connections among these three theories and their respective roles across different hypotheses are not clearly specified. Signaling theory treats BTDs as externally observable signals, while agency theory links BTDs to managerial opportunism. These logics imply different underlying assumptions, but the paper does not adequately discuss the tensions or complementarities between them.

2.Reverse causality may exist between BTDs and earnings persistence (e.g., firms expecting lower future earnings may engage in more tax planning or earnings management, affecting current BTDs), and omitted variables may confound the relationship. The current analysis primarily relies on panel fixed effects to control for unobserved heterogeneity, but fixed effects models only control for time-invariant omitted variables.

3.It is recommended that the manuscript adopt a uniform reference citation format.([2,3,19,20] [14][17].and [5],[13]) Please thoroughly check the manuscript for trivial formatting errors such as misplaced double brackets like [13]]. And standardize the first-line indentation format throughout the manuscript.

**********

what does this mean?). If published, this will include your full peer review and any attached files.

If you choose “no”, your identity will remain anonymous but your review may still be made public.

**Do you want your identity to be public for this peer review?**  For information about this choice, including consent withdrawal, please see our Privacy Policy

Reviewer #2: No

Reviewer #3: No

**********

[NOTE: If reviewer comments were submitted as an attachment file, they will be attached to this email and accessible via the submission site. Please log into your account, locate the manuscript record, and check for the action link "View Attachments". If this link does not appear, there are no attachment files.]

To ensure your figures meet our technical requirements, please review our figure guidelines: https://journals.plos.org/plosone/s/figures

You may also use PLOS’s free figure tool, NAAS, to help you prepare publication quality figures: https://journals.plos.org/plosone/s/figures#loc-tools-for-figure-preparation.

NAAS will assess whether your figures meet our technical requirements by comparing each figure against our figure specifications.

# Author comment: Author response to Decision Letter 3

**DOI:** 10.1371/journal.pone.0344270.r006

24 Sep 2026

We thank the Academic Editor and the reviewers for their careful evaluation of our revised manuscript and for their constructive comments. We have carefully addressed all remaining comments and revised the manuscript accordingly. A detailed point-by-point response is provided in our Response to Reviewers document. We believe that these revisions have further improved the clarity and rigor of the manuscript.

Attachment Submitted filename: Response_to_Editor_and_Reviewers_PONE-D-26-07956R2.docx

# Editor report: Decision Letter 3

**DOI:** 10.1371/journal.pone.0344270.r007

**Authors:** Thomas Kollruss

12 Oct 2026

When do book–tax differences matter for earnings persistence? The role of sales decline

PONE-D-26-07956R3

Dear Dr. Dung,

We’re pleased to inform you that your manuscript has been judged scientifically suitable for publication and will be formally accepted for publication once it meets all outstanding technical requirements.

Within one week, you’ll receive an e-mail detailing the required amendments. When these have been addressed, you’ll receive a formal acceptance letter and your manuscript will be scheduled for publication.

An invoice will be generated when your article is formally accepted. Please note, if your institution has a publishing partnership with PLOS and your article meets the relevant criteria, all or part of your publication costs will be covered. Please make sure your user information is up-to-date by logging into Editorial Manager at Editorial Manager® and clicking the ‘Update My Information' link at the top of the page. For questions related to billing, please contact billing support.

If your institution or institutions have a press office, please notify them about your upcoming paper to help maximize its impact. If they’ll be preparing press materials, please inform our press team as soon as possible -- no later than 48 hours after receiving the formal acceptance. Your manuscript will remain under strict press embargo until 2 pm Eastern Time on the date of publication. For more information, please contact onepress@plos.org.

Kind regards,

Thomas Kollruss, Prof. Dr.

Academic Editor

PLOS One

Additional Editor Comments (optional):

All reviewer comments have been considered. The paper is sound and can be published.

Reviewers' comments:

# Editor report: Acceptance letter

**DOI:** 10.1371/journal.pone.0344270.r008

**Authors:** Thomas Kollruss

PONE-D-26-07956R3

PLOS One

Dear Dr. Dung,

I'm pleased to inform you that your manuscript has been deemed suitable for publication in PLOS One. Congratulations! Your manuscript is now being handed over to our production team.

At this stage, our production department will prepare your paper for publication. This includes ensuring the following:

* All references, tables, and figures are properly cited

* All relevant supporting information is included in the manuscript submission,

* There are no issues that prevent the paper from being properly typeset

You will receive further instructions from the production team, including instructions on how to review your proof when it is ready. Please keep in mind that we are working through a large volume of accepted articles, so please give us a few days to review your paper and let you know the next and final steps.

Lastly, if your institution or institutions have a press office, please let them know about your upcoming paper now to help maximize its impact. If they'll be preparing press materials, please inform our press team within the next 48 hours. Your manuscript will remain under strict press embargo until 2 pm Eastern Time on the date of publication. For more information, please contact onepress@plos.org.

You will receive an invoice from PLOS for your publication fee after your manuscript has reached the completed accept phase. If you receive an email requesting payment before acceptance or for any other service, this may be a phishing scheme. Learn how to identify phishing emails and protect your accounts at https://explore.plos.org/phishing.

If we can help with anything else, please email us at customercare@plos.org.

Thank you for submitting your work to PLOS One and supporting open access.

Kind regards,

PLOS One Editorial Office Staff

on behalf of

Professor Thomas Kollruss

Academic Editor

PLOS One
